# Brainstem control of locomotion and muscle tone with special reference to the role of the mesopontine tegmentum and medullary reticulospinal systems

**DOI:** 10.1007/s00702-015-1475-4

**Published:** 2015-10-26

**Authors:** Kaoru Takakusaki, Ryosuke Chiba, Tsukasa Nozu, Toshikatsu Okumura

**Affiliations:** 1 Research Center for Brain Function and Medical Engineering, Asahikawa Medical University, Midorigaoka-Higashi 2-1, 1-1, Asahikawa, 078-8511 Japan; 2 Department of Regional Medicine and Education, Asahikawa Medical University, Asahikawa, Japan; 3 Department of General Medicine, Asahikawa Medical University, Asahikawa, Japan

**Keywords:** Postural muscle tone, Locomotor region, Pedunculopontine tegmental nucleus, Reticulospinal neurons, Behavioral state, Decerebrate cat preparation

## Abstract

The lateral part of the mesopontine tegmentum contains functionally important structures involved in the control of posture and gait. Specifically, the mesencephalic locomotor region, which may consist of the cuneiform nucleus and pedunculopontine tegmental nucleus (PPN), occupies the interest with respect to the pathophysiology of posture-gait disorders. The purpose of this article is to review the mechanisms involved in the control of postural muscle tone and locomotion by the mesopontine tegmentum and the pontomedullary reticulospinal system. To make interpretation and discussion more robust, the above issue is considered largely based on our findings in the experiments using decerebrate cat preparations in addition to the results in animal experimentations and clinical investigations in other laboratories. Our investigations revealed the presence of functional topographical organizations with respect to the regulation of postural muscle tone and locomotion in both the mesopontine tegmentum and the pontomedullary reticulospinal system. These organizations were modified by neurotransmitter systems, particularly the cholinergic PPN projection to the pontine reticular formation. Because efferents from the forebrain structures as well as the cerebellum converge to the mesencephalic and pontomedullary reticular formation, changes in these organizations may be involved in the appropriate regulation of posture-gait synergy depending on the behavioral context. On the other hand, abnormal signals from the higher motor centers may produce dysfunction of the mesencephalic-reticulospinal system. Here we highlight the significance of elucidating the mechanisms of the mesencephalic-reticulospinal control of posture and locomotion so that thorough understanding of the pathophysiological mechanisms of posture-gait disorders can be made.

## Introduction

The reticular formation, which is a core structure of the brainstem, contributes to fundamental vital activities in relation to the vigilance state of animals via the ascending and descending projections. Ascending projections modulate cortical activities via the thalamocortical networks (Steriade et al. 1993). Projections within the brainstem and those descending to the spinal cord contribute to innate motor functions such as eye movements (Hikosaka 2007; Hikosaka et al. 2000), eye–head coordination (Grantyn and Berthoz 1987; Grantyn et al. 1987), oral–pharyngolaryngeal movements (Bianchi and Gestreau 2009; Harada et al. 2005; Jean 2001), and control of posture and locomotion (Drew et al. 1986; Drew and Rossignol 1990a, b; Garcia-Rill 1986; Grillner 1981; Mori 1987; Prentice and Trevor Drew 2001; Rossignol 1996; Takakusaki 2013). In most vertebrates, reticulospinal neurons (RSNs) contribute to various types of locomotor movements such as swimming in fishes, crawling in reptiles, flying in birds, quadrupedal locomotion in higher mammals and bipedal gait in higher primates (Grillner 2011). RSNs activate neuronal circuits in the spinal cord that generate locomotor rhythm (central pattern generator; CPG) (Grillner 1981; Grillner et al. 1997; Mori 1987, 1999; Rossignol 1996; Takakusaki 2013). From an evolutionary point of view, postural control mechanisms have been developed in animals living on land, particularly for quadrupeds and bipeds, so that they can adjust their posture against the gravitational force and changes in equilibrium. Therefore, the mechanisms involved in postural muscle tone regulation and in locomotor rhythm generation are integrated so that the appropriate locomotor movements can be achieved (Mori 1987). On the other hand, RSNs with a higher activity during rapid eye movement (REM) sleep exert general inhibitory effects on brainstem and spinal cord motoneurons, resulting in muscular atonia (Chase et al. 1986; Chase and Morales 1990; Lai et al. 2010; Takakusaki et al. 2001, 2003b).

The mesencephalic and pontomedullary reticular formation (PMRF) receive direct projections from the cerebral cortex (Aravamuthan et al. 2009; Keizer and Kuypers 1989; Matsuyama and Drew 1997), limbic-hypothalamic systems (Mogenson 1991; Sinnamon 1993; Swanson and Mogenson 1981), and cerebellum (Eccles et al. 1975; Homma et al. 1995; Takahashi et al. 2014; Mori et al. 1998). In addition, efferents from the basal ganglia act projection to the brainstem via the mesopontine tegmentum (Beckstead et al. 1979; Garcia-Rill 1986; Hikosaka 2007; Saitoh et al. 2003, Spann and Grofova 1991; Takakusaki et al. 2003a, 2004b, 2011). Recently, importance of the mesopontine tegmentum, specifically the pedunculopontine tegmental nucleus (PPN), is emphasized with respect to the gait control in human. In addition to the cuneiform nucleus (CNF), the PPN is considered as a constituent of the mesencephalic locomotor region (MLR). Postural instability and gait dysfunction are the most common reason of falls for patients with Parkinson’s disease (PD) (Bloem et al. 2001; Nutt et al. 2011; Schrag et al. 2002), particularly patients with damages of cholinergic neurons in the PPN (Rinne et al. 2008; Bohnen et al. 2009). For treatment of medically refractory postural and gait abnormalities in the late stage PD, deep brain stimulation (DBS) that targets the PPN (PPN-DBS) has been performed to activate remaining cholinergic neurons. Despite promising initial results (Mazzone et al. 2008), further clinical studies showed only mild alleviation and the results were rather disappointed (Ferraye et al. 2010; Moro et al. 2010). Therefore, for a better understanding of the function of the PPN, it is necessary to elucidate how the mesopontine tegmentum is functionally organized with respect to the control of posture and gait. Specifically, following two questions should be verified. The first question is which aspects of gait control the cholinergic PPN neurons are assigned. The second is how the mesencephalic outputs orchestrate multiple reticulospinal systems so that the level of muscle tone and locomotor rhythm are appropriately regulated.

The reticulospinal tract arises from the pontine reticular formation (PRF) and the medullary reticular formation (MRF), and descends through the ventral and the ventrolateral funiculi of the spinal cord (Brodal 1981; Matsuyama et al. 1988, 1993; Sakai et al. 2009). A considerable number of RSNs send their descending axons through the neuraxis from the cervical to the lumbosacral segments, and branch off their collaterals within each segment (Matsuyama et al. 1988, 1993; Sakai et al. 2009). Such specific morphological features are functionally advantageous in orchestrating synergistic contractions of the neck, trunk, and limb musculature. However, reticulospinal effects on posture and movement differed among different experimental conditions; they depended on animal preparations employed (anesthesia versus alert) and on vigilance state (sleep-awake cycles) of animals (Chase et al. 1986; Drew and Rossignol 1990a, b; Magoun and Rhines 1946; Peterson et al. 1978, 1979). Moreover, the reticulospinal effects are strongly modified by neurotransmitters that act on the reticular formation neurons (Pace-Schott and Hobson 2002; Sakurai 2007; Takakusaki et al. 1993a, 2005). Accordingly, critical questions remain unanswered regarding functional organization of the pontomedullary RSNs with respect to the control of muscle tone and movements.

For thorough understanding of the pathophysiological mechanisms underlying posture and gait disorders, such as the PD, here we review our results with respect to the mesencephalic and reticulospinal control of postural muscle tone and locomotion in addition to findings obtained from animal experiments and clinical investigations in other laboratories. To make our interpretation and discussion more robust, this review includes unpublished findings which were obtained from experiments using decerebrate cats. Specifically, we focus on the following three issues. The first issue is the functional organization of the mesopontine tegmentum with respect to the generation of locomotion and the regulation of muscle tone. It was observed that the MLR was surrounded by the areas involved in the augmentation (dorsal) and suppression (ventral) of postural muscle tone. The second issue is the role of cholinergic PPN neurons in the integration of the locomotor rhythm and the level of muscle tone. Our findings suggest that the cholinergic neurons may simultaneously regulate the level of muscle tone and locomotor rhythm by modulating the activities of the RSNs and CPG in the spinal cord. The third question is how the reticulospinal systems are organized so that postural muscle tone and locomotion are interactively controlled. There was a gloss functional topography in the MRF in relation to the control of postural muscle tone; medullary RSNs relating to muscular atonia and those to hypertonus were located in the dorsal and ventral MRF, respectively. Moreover, locomotor signals from the MLR modulated the activities of the muscle tone-related RSNs, indicating the reticulospinal system integrates signals involved in the locomotor rhythm and muscle tone regulation. Based on these results, we propose the model of the mesencephalic and pontomedullary reticulospinal systems involved in the generation of locomotor rhythm (locomotor system) and in the control of postural muscle tone (excitatory and inhibitory systems). The pathophysiological mechanisms underlying posture-gait disorders are finally discussed with reference to the forebrain and cerebellum controls of the mesencephalic and pontomedullary reticulospinal systems.

## Functional organization of the lateral part of the mesopontine tegmentum

### Consideration of experimental procedures and limitation of approach

We start this section by demonstrating findings in our laboratory as materials for discussion of the above issues. Because the excitability of reticular formation neurons is largely influenced by anesthesia, vigilance state of the animals and inputs from the forebrain and cerebellum, we employed decerebrate cat preparations to eliminate the these factors. We employed two types of decerebrate preparations. One was the mesencephalic cat preparation in which decerebration was surgically performed at the precollicular–postmammillary level. The mesencephalic cat preparation maintains reflex standing posture. The subthalamic cat preparation was also used, in which decerebration occurs at the precollicular–premammillary level. While the subthalamic cat maintains reflex standing on a stationary surface, it also spontaneously exhibits locomotion with well-coordinated postural control, which is accompanied by largely appropriate equilibrium control (Hinsey et al. 1930). Therefore, a critical region exists between these decerebrate levels. This area is recognized as the subthalamic locomotor region (SLR), which mostly corresponds to the lateral hypothalamic area (Grillner 1981; Milner and Mogenson 1988; Mori 1987; Sinnamon 1993).

On the other hand, there is a need to consider limitation of the experimental procedures. Most data we presented in this article are entirely obtained from experiments using electrical stimulation and microinjections of neuroactive substances. Because of complexity in the structure of the reticular formation, it is really hard to know for certain what groups of neurons were being affected. Although current of electrical stimulation was reduce to less than 60 μA (usually less than 40 μA), and amount of injections of neuroactive substances was limited to less than 0.5 μl (usually less than 0.25 μl), spread of stimulation current or diffusion of substances might also change the activity of neurons other than the target neurons. This may lead to the conflicting results and conclusions. In order to avoid these risks and to obtain more precise and specific results, there is a need to use modern genetically encoded tracing methods as well as ontogenetic technologies to selectively control the activity of the target sets of neurons.

### Effects of stimulation applied to the lateral part of the mesopontine tegmentum

**Fig. 1:**
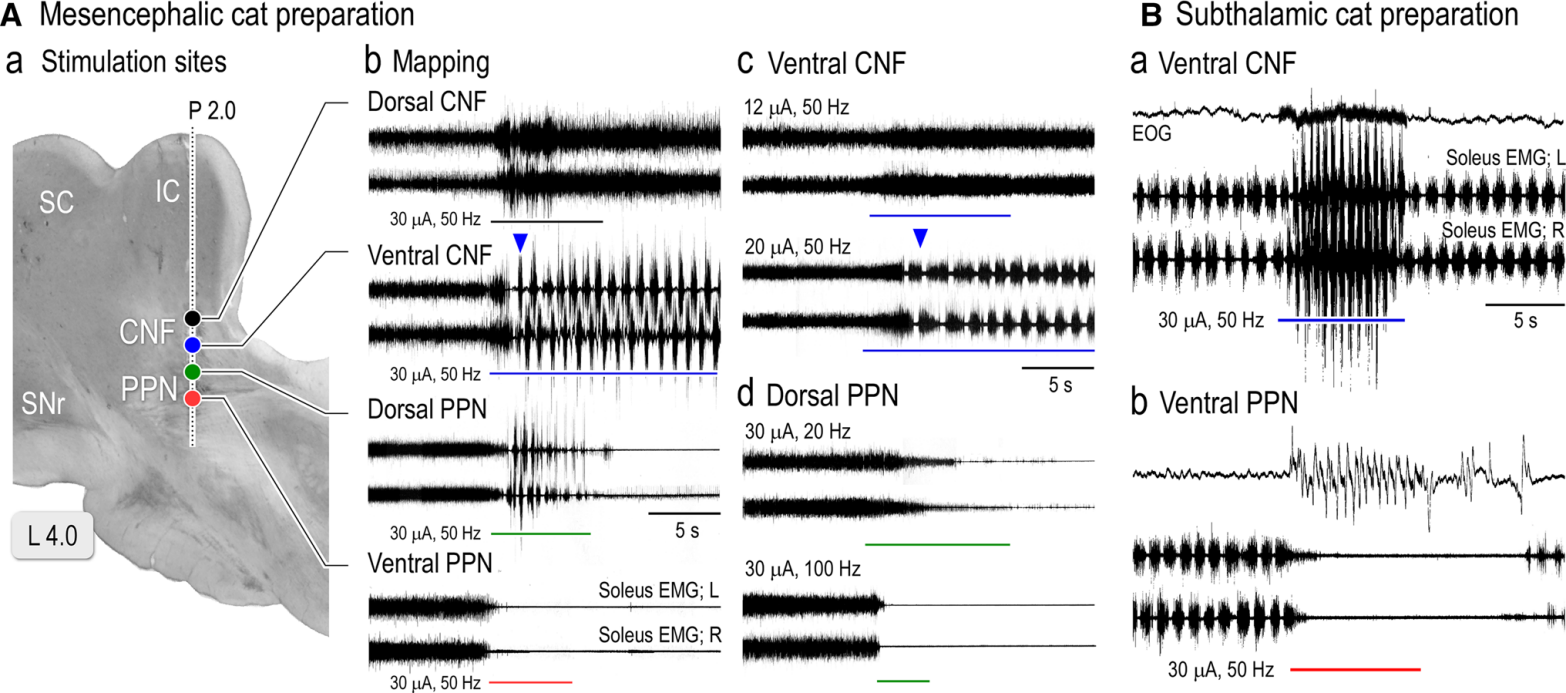
Mesopontine stimulation that evoked locomotion and suppressed muscle tone. **A** Effects of mesopontine stimulation upon soleus muscle activities in a mesencephalic cat. *a* Stimulus sites in the mesopontine tegmentum on the parasagittal plane at L 4.0. Stimuli were applied along with a track at P 2.0. Interval of each stimulation site was 1.0 mm. *b* Effects on muscle tone following stimulation to each site in (*a*). Each trace was obtained from the left (L) and right (R) soleus muscles. The *arrowhead* in (*b*) indicates treadmill onset at a speed of 0.3 m/s. The stimulation parameters were: intensity, 30 μA; frequency, 50 Hz; duration, 5–10 s. *c* Changes in soleus EMGs following the changes in the parameters of stimulation applied to the ventral CNF. Lower current of stimuli with 12 μA bilaterally increased the level of muscle tone (*upper*) and those with 20 μA elicited locomotion with reduced cycle times (*lower*). The *arrowhead* in (*c*) indicates treadmill onset at a speed of 0.15 m/s. *d* Changes in soleus EMGs following the changes in the parameters of stimulation applied to the dorsal PPN. Stimuli with either lower (20 Hz) or higher (100 Hz) frequency induced muscular atonia without any rhythmic activities. **B** Effects of stimuli applied to the ventral CNF (*a*) and ventral PPN (*b*) upon spontaneous locomotion in a subthalamic cat preparation. In each set of recording, an upper trace shows electrooculogram (EOG). Stimulation of the ventral CNF changed locomotor pattern from slow walking with cycle times of 1. 0–1.1 s to gallop with cycle times of 0.5–0.6 s. Stimulation of the ventral PPN suppressed spontaneous locomotion and induced muscular atonia, which was associated with rhythmic eye movements that had a frequency of approximately 2 Hz. *IC* inferior colliculus, *CNF* cuneiform nucleus, *PPN* pedunculopontine tegmental nucleus, *SC* superior colliculus, *SCP* superior cerebellar peduncle, *SNr* substantia nigra pars reticulata. The above findings have not been published, previously. See Takakusaki et al. (2003a, 2004c) for experimental procedures

Microstimulation applied to the mesopontine tegmentum (Fig. 1A) evoked various changes in electromyograms (EMGs) of the soleus muscles in the mesencephalic cat preparation. Repetitive stimulation applied to the dorsal part of the CNF (filled circle in Fig. 1Aa) increased the contraction of left and right soleus muscles (top traces in Fig. 1Bb). The augmentation of muscle tone lasted even after termination of the stimulation. Stimulation of the ventral CNF (blue circle in Fig. 1Aa) first evoked an alternating hindlimb loading, which developed to locomotion with a cycle time of approximately 1.0 s when the treadmill started to move (arrowhead in second traces in Fig. 1Ab). When stimuli were applied to the dorsal part of the PPN, stepping movements of the hindlimb were elicited, and they were subsequently attenuated along with a decrease in muscle tone (third traces in Fig. 1Ab). On the other hand, stimulation of the ventral part of the PPN immediately suppressed muscle tone (fourth traces in Fig. 1Ab). The muscular atonia persisted even after the stimulation.

These effects were dependent on parameters of the electrical stimulation. Stimulation with a lower current that was applied to the ventral CNF did not induce the mixed effects but induced muscle tone augmentation (upper traces in Fig. 1Ac) and locomotion with an increase in the cycle time (1.2–1.5 s; lower traces in Fig. 1Ac). In addition, stimulation of the dorsal CNF with a low (20 Hz) or high frequency (100 Hz) induced muscular atonia without any sings of the locomotor rhythm (Fig. 1Ad). However, the latency to atonia became much shorter (lower traces in Fig. 1Ad). These findings suggest that functionally different groups of neurons may exist in the area corresponding to the ventral part of the CNF and the dorsal part of the PPN. Neurons involved in the control of muscle tone and those involved in the generation of the locomotor rhythm can be functionally separated by changing the parameters of the stimulation.

In the subthalamic cat preparation, stimulation of the ventral CNF facilitated the spontaneously evoked locomotion on the treadmill; locomotor pattern was altered from walking to gallop (Fig. 1Bb). In contrast, stimulation of the ventral PPN resulted in suppression of locomotion, which was associated with rhythmic eye movements (Fig. 1Bb).

**Fig. 2:**
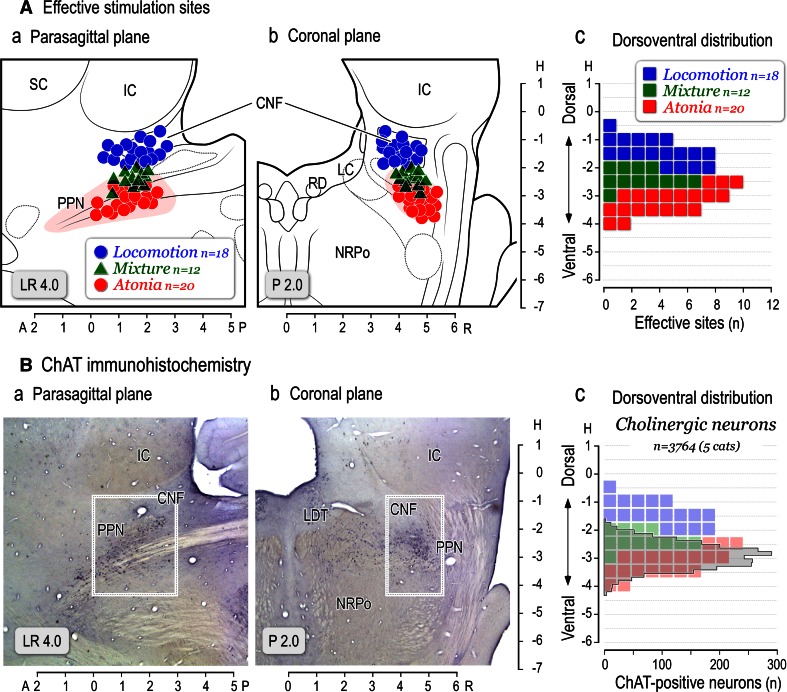
Effective sites in the mesopontine tegmentum. **A** Distribution of effective stimulation sites on the parasagittal (*a*) and coronal (*b*) planes for evoking muscular atonia (*n* = 20, *red circles*) and locomotion (*n* = 18, *blue circles*) in twenty animals. In two animals, only atonia-evoking sites were identified. Because multiple sites were involved in evoking locomotion or muscular atonia, site from which the lowest current (usually less than 20 μA) elicited each movement was determined as the optimal stimulation site. A mixture of both was obtained from twelve animals (*green triangles*). (3) Dorsoventral distribution of the optimal effective sites (*c*). **B** The distribution of cholinergic neurons stained by choline-acetyltransferase (ChAT) immunohistochemistry on the parasagittal (*a*) and coronal (*b*) planes. Effective sites in this figure include those previously published (Takakusaki et al. 2003a, 2004a, b). *c* Dorsoventral distribution of ChAT positive neurons. The ChAT positive neurons located in area demarcated by a square in each plane were counted in five animals (*n* = 3764 neurons). Note that the distribution of ChAT positive neurons is agreed well with that of stimulation sites which evoked muscular atonia (*red*) or mixture effects (*green*). *LC* locus coeruleus, *LDT* laterodorsal tegmental nucleus, *NRPo* nucleus reticularis pontis oralis, *RD* raphe dorsalis

In Fig. 2A, optimal sites for evoking locomotion (blue circles, *n* = 18), a mixture of locomotion and muscle tone suppression (green triangles, *n* = 12), and muscular atonia (red circles, *n* = 20) obtained in twenty animals were plotted on parasagittal (a) and coronal planes (b) of the brainstem. Locomotion- evoking sites were mostly located within the CNF and rostral to this nucleus. On the other hand, inhibitory sites were located in the PPN. Mixed effects were evoked between these areas. It was observed that sites for evoking locomotion and those for muscular atonia were overlapped at the dorsal part of the PPN where stimulation evoked the mixed effects. In five animals, numbers of cholinergic neurons, which were labelled by choline-acetyltransferase (ChAT) immunohistochemistry, were counted in the area demarcated by squares in the parasagittal (Fig. 2Ba) and coronal (Fig. 2Bb) planes. The dorsoventral distribution of the cholinergic neurons is agreed well with that for evoking atonia and mixed effects (Fig. 2Ac, Bc), whereas locomotion evoking sites were located in the area dorsal to the cholinergic zone of the PPN. These findings suggest followings. Firstly, functional topography may exist within the lateral part of the mesopontine tegmentum in relation to the control of locomotion (dorsal) and muscle tone (ventral). Secondly, cholinergic neurons in the PPN may largely contribute to the regulation of postural muscle tone.

### Effects of injecting atropine sulfate into the medial PRF

**Fig. 3:**
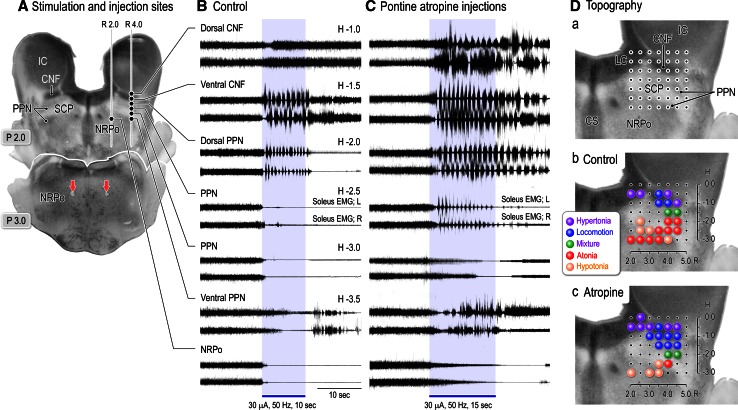
Effects of microinjections of atropine sulfate into the medial PRF upon soleus muscle EMGs. **A** Stimulation sites in the right mesopontine tegmentum and injection sites (*red arrows*) of atropine sulfate in the left and right PRF (NRPo). Injection sites were marked by electrolytic microlesions, which were made by passing DC current of 40 μA with 30 s through platinum electrode attached with a micropipette for injecting neuroactive substances. **B** Effects of stimulation applied to each site in (**A**) on left and right soleus muscle activities before atropine injections. Stimulation of the dorsal CNF induced muscle tone augmentation. While stimulation of the ventral CNF and the dorsal PPN induced locomotor rhythm, the latter was accompanied by a decrease in muscle tone. Stimulation of the PPN and NRPo immediately suppressed soleus muscle activities. Stimulation parameters were 30 μA and 50 Hz with 10 s. **C** Effects of the mesopontine stimulation after injections of atropine sulfate into the bilateral NRPo. Atropine sulfate with a concentration of 20 mM and a volume of 0.25 μl was injected through micropipette with a tip diameter less than 20 μm. Each injection was made with a rate of 0.01–0.02 μl/s. Records were obtained more than 15 min after the injections. Stimulation of the dorsal CNF bilaterally increased contractions of soleus muscles, which was more prominent in the right. Rhythmic contractions were observed in left soleus muscle. Stimulation of the ventral CNF and dorsal PPN elicited locomotion. Stimulation of the PPN (H-2.5) evoked a mixture of rhythmic activity and muscle tone suppression. Stimulation of the ventral PPN did not reduce muscle tone but evoked irregular muscle contractions. While muscular atonia was induced by stimuli applied to the PPN (H-3.0) and NRPo, latency to atonia was prolonged. Stimulation parameters were 30 μA and 50 Hz with 15 s. **D** Pontine atropine injections altered functional topography in the mesopontine tegmentum. *a* Locations of forty-nine stimulation sites, which include CNF, PPN, SCP, LC and the dorsal part of the NRPo. *b*, *c* are before and after atropine injections. Note that a number of locomotion-evoking sites increased from three to eight along with a decrease in atonia-evoking sites from nine to one. In addition, a number of hypertonus-evoking sites also increased from four to six. *CS* centralis superior, *SCP* superior cerebellar peduncle. The above findings have not been published. See Takakusaki et al. (2003a) for experimental procedures

**Fig. 4:**
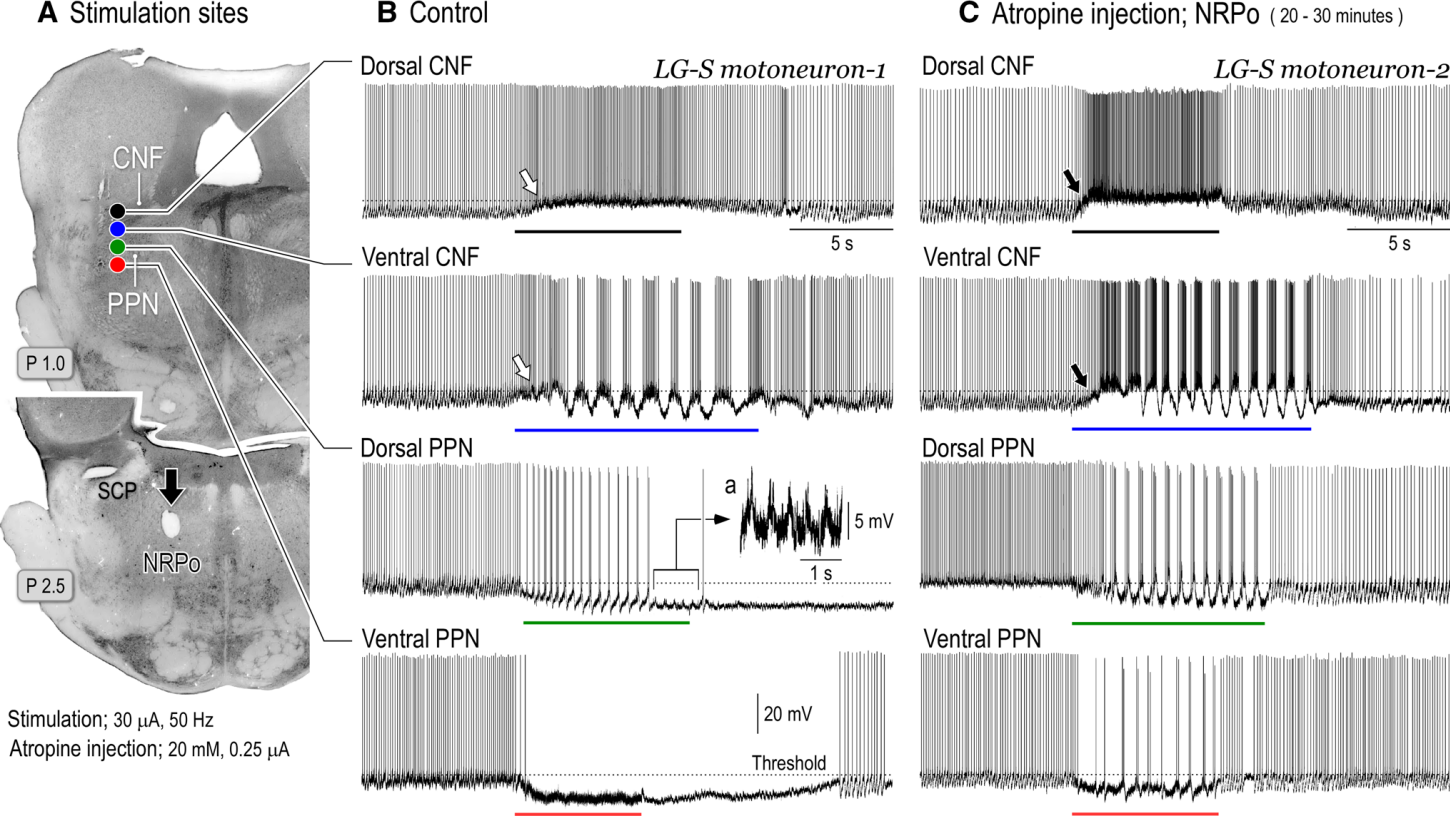
Changes in the effects of mesopontine stimulation upon hindlimb motoneurons following microinjection of atropine sulfate into the medial PRF. **A** Stimulation sites in the left mesopontine tegmentum on a coronal plane at P 1.0, and an injection site (*arrow*) of atropine sulfate in the left NRPo on a coronal plane at P 2.5. An injection site was indicated by an electrolytic microlesion. **B** Changes in intracellular activities of a lateral gastrocnemius (LG-S) motoneuron recorded from lower left lumber segment (L7) following mesopontine stimulation. Stimulation parameters were 30 μA, 50 Hz with 5–10 s. Stimulation of the dorsal CNF tonically depolarized the membrane. Stimulation of the ventral part of the CNF first depolarized the membrane and generated rhythmic membrane oscillations with a cycle time of 1.1–1.4 seconds. Stimulation of the dorsal PPN hyperpolarized the membrane potentials. The membrane hyperpolarization was followed by membrane oscillation with a cycle time of 0.4–0.5 s. In this recording, an *inset* figure *a* shows action potentials generated on the depolarizing phase of oscillation with expanded time scale. Subthreshold membrane oscillations were generated even action potentials were removed. Stimulation of the ventral PPN suppressed spontaneous firings and then induced membrane hyperpolarization which persisted even after termination of the stimulation. A line beneath each recording indicates the period of stimulation with an intensity of 30 μA and a frequency of 50 Hz. These recordings were induced by stimuli applied to each site in **A**. The *dashed line* in each recording indicates the threshold of this motoneuron. **C** Effects of the mesopontine stimulation after injections of atropine sulfate into the NRPo. Atropine sulfate consisted of a concentration of 20 mM and a volume of 0.25 μl was injected into the left NRPo. **A** Intracellular recording was made from another LG-S motoneuron between 20 and 30 min after the atropine injection. Stimulation of the dorsal and ventral CNF depolarized the membrane with faster time course (indicated by *filled arrows*) compared to control (indicated by *open arrows*). The ventral CNF stimulation subsequently generated membrane oscillations with a cycle time of 0.8–0.9 s. Stimulation of the dorsal PPN induced membrane hyperpolarization which was accompanied by fast membrane oscillations with a cycle time of approximately 0.6 s. The oscillation was not terminated during the period of stimulation. Stimulation of the ventral PPN did not suppress spontaneous firings of the motoneuron. Input resistance of the LG-S motoneuron-1 and 2 were 1.5 and 1.4 MΩ, respectively. The above findings have not been published, previously. See Takakusaki et al. (2004a, 2011) for experimental procedures

**Fig. 5:**
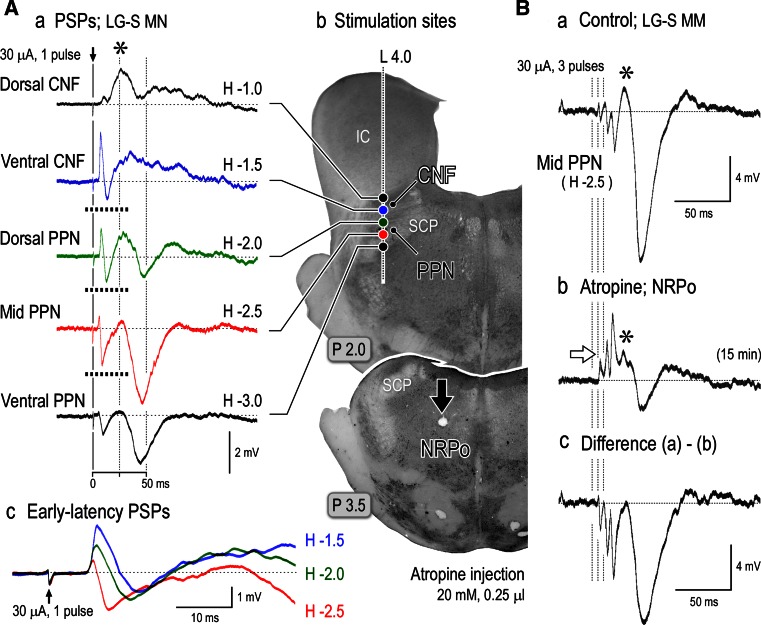
Postsynaptic potentials (PSPs) in hindlimb motoneurons evoked by mesopontine stimulation. **A** *a* PSPs evoked by stimuli (1 pulse and 30 μA) applied to the left mesopontine tegmentum along with a track of L 4.0 on a coronal plane at P 2.0 (*b*). Recordings were obtained from an LG-S motoneuron. Each record was averages of 16 sweeps. The dorsal CNF evoked an EPSP (an *asterisk*) with a peak latency of approximately 25 ms (middle-latency EPSP). The middle-latency EPSPs were also evoked by stimuli applied to the ventral CNF and dorsal PPN. IPSPs with a peak latency of 45–50 ms (late-latency IPSP) were evoked by stimuli applied to the dorsal, mid, and ventral PPN. Stimuli applied to the ventral CNF, dorsal PPN, and mid PPN evoked a mixture of EPSPs and IPSPs in an early latency, which are illustrated in *c* with expanded time scale. The minimum latency of the early EPSP was 6.8 ms and that of the early IPSP was 8.0 ms. **B** Changes in PPN-induced PSPs following injection of atropine sulfate into the NRPo. Atropine sulfate (20 mM and 0.25 μl) was injected into the left NRPo which was indicated by an *arrow* on a coronal plane at P 3.5 (*b* in **A**). *a* Before atropine injection, short train pulse of stimuli (three pulses and 30 μA) applied to the mid PPN evoked early-latency IPSPs followed by middle-latency EPSP (denoted by *asterisk*) and late-latency IPSP with a peak amplitude of 13.5 mV. *b* 15 min after the atropine injection, amplitude of the late-IPSP was reduced to 2.5 mV. Moreover, early-IPSPs were no more observed. Instead, early-latency EPSPs became evident. On the other hand, amplitude of the middle EPSP was nearly unaffected. *c* Difference of PPN-induced PSPs before and after atropine injection. Only IPSP components were attenuated by the atropine injection. The above findings have not been published, previously. See Takakusaki et al. (2011) for experimental procedures

Then, how do the cholinergic neurons in the PPN contribute to the control of muscle tone and locomotion? Because the medial PRF is one of the major targets of cholinergic projections from the PPN (Lai et al. 1993; Mitani et al. 1988; Semba 1993), examinations were made to determine whether the effects by the mesencephalic stimulation were modulated by the excitability of cholinoceptive PRF neurons. For this, we injected atropine sulfate, a cholinergic muscarinic antagonist, into the medial PRF, and examined the effects of mesopontine stimulation upon postural muscle tone and locomotion (Fig. 3), intracellular activities of hindlimb motoneurons (Fig. 4), and postsynaptic potentials (PSPs) in the motoneurons (Fig. 5).

#### Changes in postural muscle tone and locomotion

Effects of the mesopontine stimulation upon soleus EMGs, which are shown in Fig. 3B, well resembled to those shown in Fig. 1A. Stimulation of the dorsal CNF bilaterally increased the level of muscle tone (top traces in Fig. 3B), and that of the ventral CNF evoked locomotion (second traces). A mixture of locomotion and muscle tone suppression was evoked from the dorsal PPN (third traces). Stimuli applied to the PPN and to the ventral PPN resulted in muscular atonia (fourth and fifth traces) and hypotonia (bottom traces), respectively. The identical stimuli applied to the medial PRF, which corresponds to the nucleus reticularis pontis oralis (NRPo), also evoked muscular atonia (bottom traces in Fig. 1B). Totally effects from 49 sites on the coronal plane of the mesopontine tegmentum were examined in this animal (Fig. 1Da). Locomotory activities were evoked from the medial and ventral part of the CNF (*n* = 3, blue circles; locomotion-evoking sites). Stimuli of the dorsal and lateral parts of the CNF in addition to the locus coeruleus (LC) increased the level of muscle tone (*n* = 4, violet circles; hypertonus- evoking sites). In contrast, muscular atonia (*n* = 9, red circles; atonia-evoking sites) and hypotonia (*n* = 4, orange circles; hypotonus evoking sites) were induced by stimuli applied to the PPN and NRPo. A mixture of muscle tone suppression and locomotor rhythm was evoked by stimuli applied to the dorsal part of the PPN (*n* = 2, green circles).

Effects of the mesopontine stimuli were markedly altered by injections of atropine sulfate into the left and right NRPo (downward arrows in Fig. 1A). First, the CNF-induced locomotor activities were facilitated; the cycle time of the locomotion was reduced from 1.0 s (control) to 0.7 s after atropine administration (second traces in Fig. 1C). In addition, stimulation of the dorsal PPN elicited locomotion (third traces). Stimuli applied to the dorsal CNF (top traces) and to the PPN (fourth traces) also generated rhythmic activities. Second, suppressive effects from the PPN and NRPo were greatly attenuated; the latency to muscular atonia was increased (fifth and bottom traces). Stimulation of the ventral PPN even evoked movements (sixth traces). Consequently, as shown in Fig. 3Dc, pontine atropine injections increased the numbers of locomotion-evoking sites (*n* = 8) and hypertonus-evoking sites (*n* = 6). Instead, a number of atonia-evoking sites (*n* = 1) was reduced. Based on the above findings, following suggestions can be made. First, the cholinergic PPN-PRF projection is involved in the regulation of postural muscle tone and locomotor rhythm. Second, functional topographical organization may exist in the mesopontine tegmentum with respect to the regulation of postural muscle tone and locomotion. Third, the functional organization is altered depending on the activity of the cholinergic PPN-PRF projection.

#### Changes in excitability of motoneurons

Locomotor rhythm is generated by central pattern generator (CPG), which is composed of interneuronal networks in the spinal cord (Grillner 1981). The activity of the CPG is observed as rhythmic membrane oscillations in motoneurons during locomotion (Jordan et al. 2008; Shefchyk and Jordan 1985). To examine the supraspinal control of locomotor rhythm in motoneurons, the decerebrate cat was immobilized to remove the influence of sensory afferents. Findings are shown in Fig. 4. In a lateral gastrocnemius-soleus (LG-S) motoneuron, membrane potential was depolarized during the period of stimulation (top trace in Fig. 4B) applied to the dorsal CNF (filled circle in Fig. 4A). Stimulation of the ventral CNF (blue circle) first depolarized the membrane and then produced a sequence of rhythmic membrane oscillations with a cycle time of 1.1–1.4 s (second trace in Fig. 4B). Action potentials were generated on the depolarizing phase of the oscillations. Stimulation of the dorsal PPN (green circle in Fig. 4A) induced membrane hyperpolarization, which was associated with rhythmic firings of the motoneuron with intervals between 0.3 and 0.5 s (third trace in Fig. 4B). Even after termination of the firing, membrane oscillations remained (inset in Fig. 4Ba). The oscillations had a cycle time of appropriately 0.5 s. Stimulation of the ventral part of the PPN (red circle in Fig. 4A) stopped firing and hyperpolarized the membrane (bottom trace in Fig. 4B). These changes in the excitability of the motoneuron well reflect the changes in soleus EMGs shown in Fig. 1B. Following changes were observed after atropine injection into the medial PRF (downward arrow in Fig. 4A). First, stimulation of the dorsal and ventral CNF depolarized the membrane with faster time course (arrows in the first and second traces in Fig. 4C) compared to the control (open arrows in Fig. 4B). Second, locomotor activities were facilitated; the CNF-induced locomotion had oscillations with cycle times of 0.8–0.9 s (second trace in Fig. 4C). In addition, stimulation of the dorsal PPN continuously generated locomotor activity which was accompanied by rhythmic firing of the motoneuron (third traces in Fig. 4C). Third, the inhibitory effect evoked from the PPN was attenuated; stimulation of neither the dorsal nor ventral PPN blocked generation of action potentials (third and bottom traces in Fig. 4C).

#### Changes in postsynaptic potentials evoked by mesencephalic stimulation

Single pulse stimulation was applied to the mesopontine tegmentum (Fig. 5Ab), and PSPs evoked from each site were recorded (Fig. 5Aa). Mesopontine stimulation usually evoked a mixture of excitatory (EPSPs) and inhibitory postsynaptic potentials (IPSPs) with various latencies in hindlimb motoneurons. Stimulation of the dorsal CNF evoked EPSPs with a peak latency of around 25 ms (middle EPSP). Stimulation of the ventral CNF evoked a mixture of EPSP and IPSP within 20 ms (early-PSPs), which was followed by the middle EPSP. Stimulation of the mid and ventral part of the PPN evoked a sequence of EPSP and IPSP in the early latency, and they were followed by large IPSPs with a peak latency of 45 ms (late-IPSP). Stimulation of the dorsal PPN evoked a mixture of EPSPs and IPSPs whose time course was similar to those evoked from both the ventral CNF and PPN. The amplitude of the middle EPSP was the largest when the dorsal CNF was stimulated, and it was gradually reduced by stimuli applied to the ventral sites. Conversely, the late-IPSP was the most prominent when mid PPN was stimulated, and the amplitude was reduced by stimuli applied to either the dorsal or ventral site. Accordingly, neuronal mechanisms involved in the middle-EPSP and late-IPSP may contribute to the increase and decrease in the level of postural muscle tone, respectively. On the other hand, a mixture of the early-EPSP and IPSP was effectively evoked by stimuli applied to the ventral CNF and dorsal PPN (third and fourth traces). These sites well correspond to locomotion evoking area. The early-PSPs are superimposed in Fig. 5Ac with an expanded time scale; the minimum latency of the early-EPSPs was 6.8 ms (blue and green traces), and that of the IPSP (red trace) was approximately 1 ms later than the EPSPs. Therefore, the early-PSPs are possibly mediated by fast-conducting glutamatergic RSNs and spinal interneurons that are involved in the generation of locomotor rhythm (Jordan et al. 2008; Shefchyk and Jordan 1985).

Examinations were further made to elucidate whether cholinergic PPN neurons were involved in the generation of these PSPs. Triple pulses of stimuli applied to the PPN (red circle in Fig. 4Ab) evoked early-IPSPs which were followed by the middle-EPSP (asterisk in Fig. 5Ba) and late-IPSP (Fig. 4Ba). An injection of atropine into the PRF (filled arrow in Fig. 5Ab) greatly reduced the amplitude of the early- and late-IPSPs. On the other hand, the early-EPSP became evident (open arrow Fig. 4Bb). However, the middle-EPSP (asterisk in Fig. 5Bb) was unaffected. The difference of these PSPs (Fig. 5Bc) between before and after atropine injection reveals that the cholinergic PPN-PRF projection is involved in evoking both the early- and late-IPSPs. On the other hand, the early- and middle-EPSPs may be ascribed to the activation of non-cholinergic neurons in the mesopontine tegmentum.

Consequently findings in Figs. 3, 4 and 5 lead us following suggestions. (1) Cholinergic neurons in the PPN are involved in muscle tone suppression. (2) Non-cholinergic neurons in the dorsal CNF are involved in muscle tone augmentation. (3) Both the cholinergic and non-cholinergic neurons in the mesopontine tegmentum may be required to generate the locomotor rhythm.

## Neuronal components of the MLR

Findings in animal experiments and those in clinical studies generally suggest the importance of cholinergic neurons in the PPN in the control of posture and gait. Moreover the damage of the cholinergic neurons is associated with frequent falling in PD (Bohnen et al. 2009; Karachi et al. 2010; Rinne et al. 2008). However, it has not been well understood how cholinergic PPN neurons are involved in the mechanisms of controlling posture and gait.

### Studies in quadruped animals

Knowledge on the control of posture and gait largely relays on findings obtained in quadruped animals. The MLR was first discovered in decerebrate cats by Shik et al. (1966), and this functional region appears to be present in all classes of vertebrates (Grillner et al. 1997). However, anatomical constituents of the MLR remain a matter of debate, particularly relative to the PPN and CNF.

#### Anatomical constituents of the MLR

The PPN is located in the ventrolateral portion of the caudal mesencephalic reticular formation (Olszewski and Baxter 1954), and is composed of a heterogeneous population of neurons containing gamma-amino-butyric acid (GABA), and glutamate in addition to acetylcholine (ACh) (Clements and Grant 1990; Ford et al. 1995; Lavoie and Parent 1994; Mena-Segovia et al. 2008, 2009; Saitoh et al. 2003; Takakusaki et al. 1996). Cells in the PPN are also characterized by other neuronal markers, including calcium-binding proteins and neuropeptides (Fortin and Parent 1999; Vincent 2000; Vincent et al. 1983). The cholinergic neurons serve to delineate PPN boundaries, identifying a pars compacta and a pars dissipata (Mesulam et al. 1989). The CNF lies dorsal to the PPN and ventral to the superior and inferior colliculi. However, it is often difficult to clearly distinguish the boundary of these nuclei.

Garcia-Rill et al. (1987, 2011) refined the anatomical boundary of the MLR in rats to a restricted region of the mesopontine tegmentum including the PPN, and they used markers of cholinergic neurons to convincingly demonstrate that locomotion was induced by the activation of cholinergic neurons within the PPN (Skinner et al. 1990a, b, c). Similarly, Mogenson et al. (Brudzynski and Mogenson 1985; Milner and Mogenson 1988; Brudzynski et al. 1993) ascribed the effects of electrical stimulation, drug injection and lesions to the actions of the PPN in evoking locomotion in rats. Since then, cholinergic PPN neurons have been widely considered a key element of the MLR. However, the same procedures in the cat and rat also produced a numbers of results showing that the effective sites were mainly located in and around the CNF including a vicinity in the PPN (Amemiya and Yamaguchi 1984; Brudzynski et al. 1986; Coles et al. 1989; Depoortere et al. 1990; Mori et al. 1989; Shik et al. 1966; Shik and Orlovsky 1976; Sterman and Fairchild 1966; Takakusaki et al. 2003a). In addition, the activity-dependent expression of *c*-*Fos* following treadmill locomotion rats was not detectable in the PPN but in the CNF (Jordan 1998). Similarly, 2-deoxyglucose labeling revealed an increased activity only in the CNF following MLR-evoked locomotion in cats (Shimamura et al. 1987). A recent study by Gut and Winn (2015) shows that complete lesions of PPN did not cause any gait deficits in the rat, throwing doubt on question as to the status of PPN as a motor control structure. Paradoxically, the PPN is labeled with *c*-*Fos* during rapid REM (Shiromani et al. 1992, 1995, 1996), indicating that the PPN rather contributes to the generation of REM sleep (Datta 2002; Ford et al. 1995; Jones 1991, 2005; Koyama and Sakai 2000; Lai et al. 1993; Mitani et al. 1988; Semba 1993).

Our findings using decerebrate cat preparation revealed that optimal sites for evoking locomotion, i.e., MLR, are mostly located in the ventral part of the CNF and the vicinity in the dorsal part of the PPN (Fig. 2; Takakusaki et al. 2003a). Moreover, neurons in the dorsal part of the CNF and the ventral of the PPN may contribute to the increase and decrease in the level of postural muscle tone, respectively. Therefore, the MLR is, functionally, surrounded by the areas involved in the augmentation and suppression of postural muscle tone. The atonia induction zone well corresponds to PPN pars compacta where abundant cholinergic neurons are located (Fig. 2). Importantly, such a functional organization of the mesopontine tegmentum may be altered depending on excitability of the cholinergic neurons in the PPN and cholinoceptive neurons in the medial PRF. If the excitability of these neurons is higher, the excitation of the mesencephalic neurons may reduce muscle tone, whereas it may increase the level of muscle tone and/or elicit locomotion when excitability of these neurons is lower.

It is of worth to note that the effects by stimulating the MLR can be separated by changing parameters of electrical stimuli. Based on the findings in Fig. 1, neurons that are involved in the locomotor rhythm may have a threshold higher than those which contribute to the augmentation of muscle tone (Fig. 1Ac). Additionally, they may optimally respond to the stimulation with a frequency of around 50 Hz (between 20 and 100 Hz, Fig. 1Ad). In other words, neurons responsible for muscle tone suppression may respond to the stimuli with wide frequency ranges. Accordingly, the MLR may be composed of functionally different groups of neurons; neurons which contribute to the control of muscle tone and those to the generation of locomotor rhythm are intermingled.

#### Role of cholinergic and non-cholinergic neurons in the PPN

A large body of findings in quadruped animals indicates that cholinergic neurons in the PPN may not be principal neural elements of the MLR. Nonetheless, our studies suggest that cholinergic PPN neurons may play a crucial role in the control of posture and locomotion. Specifically, cholinergic neurons are critically involved in the suppression of muscle tone in addition to the modulation of the locomotor rhythm (Figs. 3, 4, 5, Takakusaki et al. 2003a, 2004a, 2011). We propose that the muscle tone inhibitory system arising from cholinergic neurons in the PPN via the cholinoceptive PRF neurons and the medullary RSNs regulates the excitability of spinal cord interneurons that comprise CPG as well as that of motoneurons. Therefore, the locomotor rhythm and postural muscle tone can be simultaneously modulated (see section “The inhibitory system”, Fig. 15).

A considerable population of cholinergic PPN neurons contains glutamate (Lavoie and Parent 1994). Then, what is the possible role of the glutamatergic neurons? Jordan et al. (2008) suggest that non-cholinergic neurons in the MLR area elicit locomotion via fast-conducting RSNs in the medial MRF. Findings in this article also suggest that non-cholinergic neurons contribute to both the regulation of locomotor rhythm and the level of postural muscle tone. This suggestion is based on following observations; pontine atropine injections facilitated locomotory activities evoked from the ventral CNF and enhanced excitatory effects from the dorsal CNF despite of attenuation of the PPN-induced inhibitory effects (Figs. 3, 4). Similarly, EPSP components with the early and middle latencies were not attenuated by the pontine atropine injection (Fig. 5). The former and the latter may contribute to the generation of locomotor rhythm and muscle tone augmentation, respectively (see section “Changes in postsynaptic potentials evoked by mesencephalic stimulation”).

The PPN also has descending projections to the medioventral part of the MRF (Nakamura et al. 1989) and spinal cord (Skinner et al. 1990a, b, c; Spann and Grofova 1989). There is a suggestion that both cholinergic and non-cholinergic (glutamate and substance P) projections to the medioventral MRF are likely involved in the initiation of locomotion (Kinjo et al. 1990; Skiner et al. 1990a, b, c). It should be noted that substance P containing neurons in the mesopontine tegmentum are severely damaged in patients of PD (Gai et al. 1991). PPN neurons with projections to the spinal cord are possibly non-cholinergic in nature (Skinner et al. 1990a, b, c). Role of the spinal projecting neurons has not been examined. A recent study using in rats by Sherman et al. (2015) showed that glutamatergic RSNs just medial to the PPN contributed to the locomotor behaviors.

### Findings in human and non-human primates

A report by Masdeu et al. (1994) is the first to suggest the presence of MLR in human; a patient with a lesion in the pontomesencephalic region, which included the CNF and PPN, could not stand and walk. Since then, patients with lesions in the corresponding area have been reported to show ataxic gait difficulties (Midbrain ataxia; Hathout and Bhidayasiri 2005). Recent imaging studies in human demonstrate the role of MLR area in the control of posture and gait (Jahn et al. 2008a, b; Karachi et al. 2012). Particular interesting findings are obtained by Karachi et al. (2012) who showed that the CNF and PPN had different role in the locomotor control; the CNF and the dorsal part of the PPN may control motor aspects of locomotion, whereas the ventral part of the PPN may be involved in integrating sensory information. In healthy subjects, these authors revealed significant correlations between the activity of the MLR and the speed of imagined gait; a faster imagined gait activated a discrete cluster within the MLR (Karachi et al. 2010).

Postmortem studies in patients with PD demonstrate that about 50 % of the large cholinergic neurons in the pars compacta of the PPN are degenerated (Bohnen and Albin 2011; Hirsch et al. 1987; Jellinger 1988; Zweig et al. 1989). PD patients with cholinergic cell loss in the PPN showed more severe motor disabilities with gait and posture, which were associated with 1-3,4-dihydroxy-phenylalanine (DOPA)-resistant akinesia (Bohnen et al. 2009; Karachi et al. 2010; Rinne et al. 2008). Subsequent postmortem study in PD patients established a correlation between the occurrence of falls and freezing and the loss of cholinergic PPN neurons. However, the degree of neuronal loss in the CNF was not significantly different between fallers and non-fallers in PD patients (Karachi et al. 2010). In PD patients, individual neurons in the dorsal PPN increased their firing rates with increased stepping frequency (Piallat et al. 2009). Moreover, gait speed in PD patients was correlated with a power of alpha-oscillations (7–10 Hz) of field potentials recorded from the PPN area (Thevathasan et al. 2012). The frequency of the oscillations corresponds to fundamental firing rates of cholinergic PPN neurons in in vitro (Takakusaki et al. 1997). These clinical studies in PD patients suggest that cholinergic neurons of the PPN are critically involved in the control of posture and gait.

To verify the above possibility, Karachi et al. (2010) demonstrated that selective lesions of PPN neurons using a specific neurotoxin without damages of nigrostriatal dopaminergic neurons led to prominent deficits in posture and gait of the monkey. Moreover, the loss of PPN cholinergic neurons was correlated with balance deficits in aged monkey with a damage of the dopamine (DA) neurons by 1-methyl-4-phenyl-1,2,3,6-tetrahydropyridine (MPTP) intoxication. In addition, a combination of bilateral PPN lesions and damages of the DA neurons by MPTP induced a dual effect; there was an improvement of hypokinesia contrasting with a worsening of DOPA-unresponsive gait and balance dysfunction (Grabli et al. 2013). These results clarify the pathophysiology of DOPA-resistant deficits of posture and gait in the advanced PD and highlight the key role of the PPN cholinergic lesion in these symptoms.

Then, the critical question is as to how the loss of cholinergic PPN neurons leads gait deficiency. One of the possible interpretations would be that a damage of the descending pedunculopontine-reticular (PPN-PRF) cholinergic projections may reduce the capability of muscle tone regulation, as can be considered in the above, resulting in impairments of posture-gait disturbance. Alternatively, a damage of the ascending pedunculopontine-thalamic cholinergic projections may disturb integration of sensory signals which contributes to the postural control. Indeed, a reduction of the thalamic cholinergic innervation in patients with PD has no cognitive and motor impairments but exhibits an increase in postural sway speed (Müller et al. 2013).

### Cholinergic systems participating in gait control via their role in modulating attention

Cholinergic neurons in the PPN have ascending projections to the non-specific thalamocortical system which stimulates cortical activation (Hallanger et al. 1987; Jones 2005; Pahapill and Lozano 2000; Steriade et al. 1993; Winn 2008). To a lesser degree, PPN cholinergic neurons also project through the ventral extra-thalamic pathway to the posterior hypothalamus and basal forebrain (BF) in addition to the striatum (Dautan et al. 2014) and midbrain DA neurons (Futami et al. 1995; Mena-Segovia et al. 2008; Takakusaki et al. 1996). Accordingly, the PPN integrates diverse information to produce a variety of behavioral expression (Alam et al. 2011; Benarroch 2013; Takakusaki et al. 2008). Therefore, motor, cognitive and sleep disturbances in PD patients can be largely attributed to the damage of the PPN that has such unique anatomical and physiological position (Pahapill and Lozano 2000; Parent and Descarries 2008; Winn 2008).

Cholinergic BF projections to the cerebral cortex are necessary for attentional performance (Hasselmo and Sarter 2011). This nucleus also has projections to the PPN and LDT (Semba and Fibiger 1992). Of particular interest is that the degeneration of the BF cholinergic system in patients of PD is often more severe than in Alzheimer’s disease (Bohnen et al. 2003). Degeneration of BF cholinergic projections correlates with the reduced walking speed in patients of PD (Bohnen et al. 2012). Although the exact mechanism of the cortical cholinergic system in the control of gait is still unclear, slower gait may be related to decreasing cognitive processing abilities during ambulation (Allcock et al. 2009; Bohnen et al. 2013; Woollacott and Shumway-Cook 2002). This may also explain why patients in PD with more severe posture-gait instability have a high risk of developing dementia (Müller and Bohnen 2013). Parikh et al. (2013) recently showed that presynaptic choline transporter in mice regulated the sustained release of ACh in the cerebral cortex so that cognitive and attentive tasks were maintained.

A loss of cholinergic neurons in the BF and PPN in addition to the damage of midbrain DA neurons associates with fallers in PD (Bohnen and Albin 2011; Bohnen et al. 2009; Müller and Bohnen 2013). Then, which is the primary source of falls? To answer this question, Kucinski and Sarter (2015) made challenging experiments in the rat to determine the effects of selective cholinergic PPN lesions in combination with striatal DA loss or BF cholinergic cells loss as well as losses in all three regions. Because the performance of rats with losses in all 3 regions was not more severely impaired than following combined BF cholinergic and striatal DA lesions, they conclude that the BF cholinergic-striatal disruption of attentional-motor interactions may be a primary source of falls.

### Consideration of the DBS that targets the PPN area

DBS targeting the PPN (PPN-DBS) has been performed to alleviate freezing of gait in PD patients with the aim of stimulating remaining cholinergic neurons (Benarroch 2013; Hamani et al. 2007, 2011, Pereira et al. 2011; Stefani et al. 2007). The first studies using DBS in advanced PD patients concluded that low-frequency stimulation of the PPN could be effective to control freezing of gait and falls (Mazzone et al. 2005, 2008). However, further clinical studies concluded that freezing of gait were mildly improved by PPN-DBS but the overall results were rather disappointing (Ferraye et al. 2010; Moro et al. 2010). These results emphasize the need to determine the optimal surgical target (Alam et al. 2011; Karachi et al. 2012; Mazzone et al. 2011). Ferraye et al. (2010) suggest that the most suitable targets are located slightly posterior to the PPN pars compacta, probably in the ventral part of the CNF where stimulation-induced locomotion has been reported in animals (Takakusaki et al. 2003a). This area possibly corresponds to the subcuneiform nucleus as described by Alam et al. (2011). Karachi et al. (2012) also suggest that it may be the case that treating PD patients suffering from failure of gait initiation versus falling may require specifically targeting the CNF and the dorsal part of the PPN, respectively.

The degeneration of cholinergic neurons also relates to the higher incidence of REM sleep behavior disorder symptoms in patients with PD (Lima 2013; Müller et al. 2015). Peppe et al. (2012) reported that stimulation of the PPN not only improved nighttime sleep, unlike DBS targeting to the subthalamic nucleus (STN), but also ameliorated daytime sleepiness. On the other hand, some PD patients who received PPN-DBS are reported to enter either non-REM sleep or REM sleep episode (Arnulf et al. 2010). Such a heterogeneous outcome on the effects of the PPN-DBS on sleep regulation may also relate to the anatomical and physiological complexity in the mesopontine tegmentum.

Findings obtained in the decerebrate cat may be available for the interpretation of the variety of the effect induced by the PPN-DBS. Namely, effects of the mesencephalic stimulation depend on not only the site of stimuli but also parameters of electrical stimulation and the excitability of neurons in the PPN and PRF (Figs. 1, 2, 3, 4, 5). Specifically, considerable attention should be placed on the importance of cholinergic and monoaminergic neurons in the control of the excitability of PMRF neurons in relation to vigilance states (Chase and Morales 1990; Jones 2005; Pace-Schott and Hobson 2002). Possibly, a PPN-DBS may not reduce muscle tone but improves gait capability during wakefulness where the excitability of cholinergic PPN neurons and cholinoceptive PRF neurons is likely less active. In contrast, even for the identical stimulus, it may reduce postural muscle tone and induce a state liable to enter the REM sleep if the excitability of these neurons is high. Because the excitability of the PRF is modulated by the interaction between the cholinergic and monoaminergic systems (Takakusaki et al. 1993a, 1994), it is critical to determine how such an interaction occurring at the PRF alters the activity of the RSNs which are involved in the control of postural muscle tone and locomotion. These issues are considered in the following sections.

## Reticulospinal control of postural muscle tone and locomotion

**Fig. 6:**
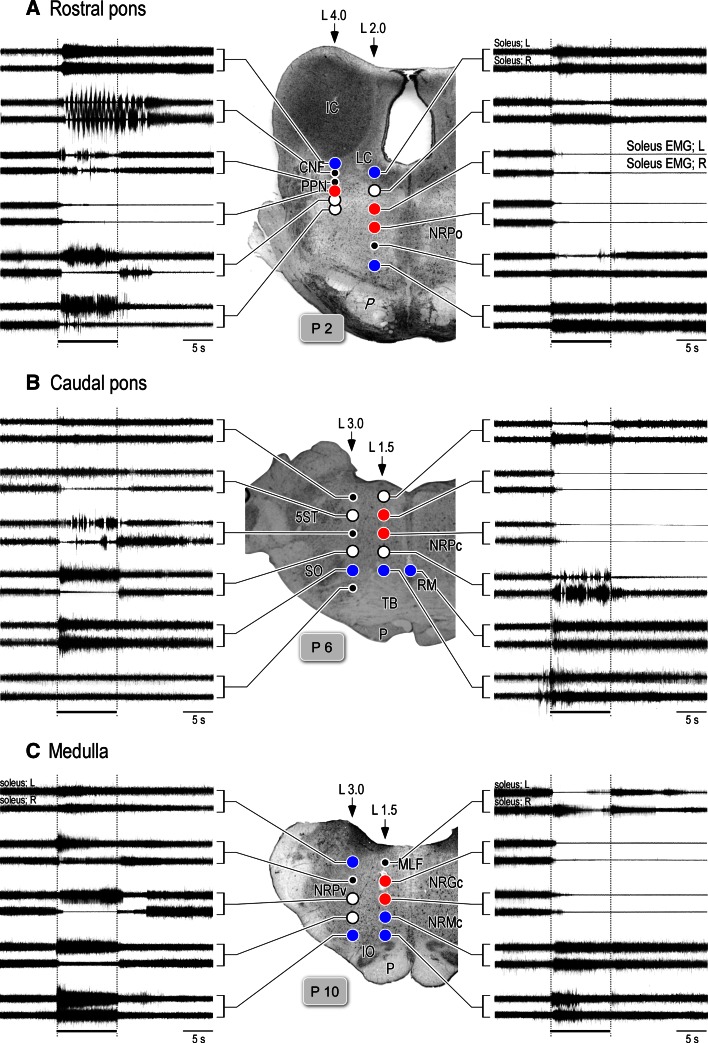
Pontomedullary reticular formation (PMRF) stimulation-induced changes in electromyograms (EMGs) acquired from both soleus muscles. **a**–**c** Stimulation sites in the rostral pons (**a**), caudal pons (**b**) and medulla (**c**), and the effects of electrical stimulation (50 Hz and 40 μA, lasting for 10 s) applied to each site upon bilateral contractions of the soleus muscle. The results in **b**–**d** were obtained from different animals. In each cat, stimuli were applied at 0.5 mm intervals in the dorsoventral and mediolateral directions. This figure shows the effects on the rostral pons when stimuli were applied along the L2.0 and L4.0 tracks (**a**). For the caudal pons (**b**) and medulla (**c**) traces, stimuli were applied along the L1.5 and L3.0 tracks. Sites from which the stimulation evoked bilateral suppression and bilateral augmentation are indicated by *red* and *blue circles*, respectively. *Open circles* indicate sites from which the delivered stimulation evoked tegmental reflexes. Stimulation applied to the medial sites evoked the contraction in contralateral (*right*) muscle and relaxation in the ipsilateral (*left*) muscle. However, stimulation of the lateral sites evoked ipsilateral (*left*) contraction and contralateral (*right*) relaxation. The above findings have not been published, previously

**Fig. 7:**
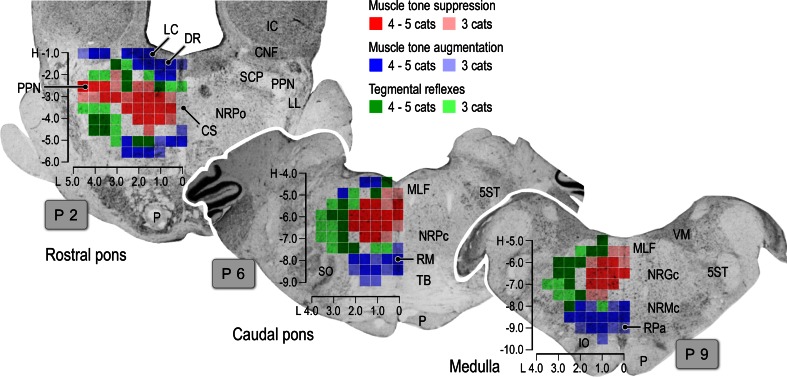
Stimulation sites that were effective in changing the posture of decerebrate cats. Results obtained from five animals are superimposed on representative coronal planes of the rostral pons, caudal pons, and medulla. Sites from which either suppression (*red*), augmentation (*blue*) or tegmental reflexes (*green*) was elicited in more than three out of five animals are marked. Sites from which the stimulation-induced postural changes in more than four animals are indicated by *darker colored squares*; conversely, *light colored squares* indicate that the postural changes were induced in three animals. Please see text for further explanation. *LL* lateral lemniscus, *CS* nucleus centralis superior, *DR* dorsal raphe, *P* pyramidal tract, *MLF* medial longitudinal fasciculus, *5ST* spinal trigeminal tract, *NRPc* nucleus reticularis pontis caudalis, *TB* trapezoid body, *RM* nucleus raphe magnus, *SO* superior olive, *VM* medial vestibular nucleus, *NRGc* nucleus reticularis gigantocellularis, *NRMc* nucleus reticularis magnocellularis, *RPa* nucleus raphe pallidus, *IO* inferior olive. The above data have not been published previously

**Fig. 8:**
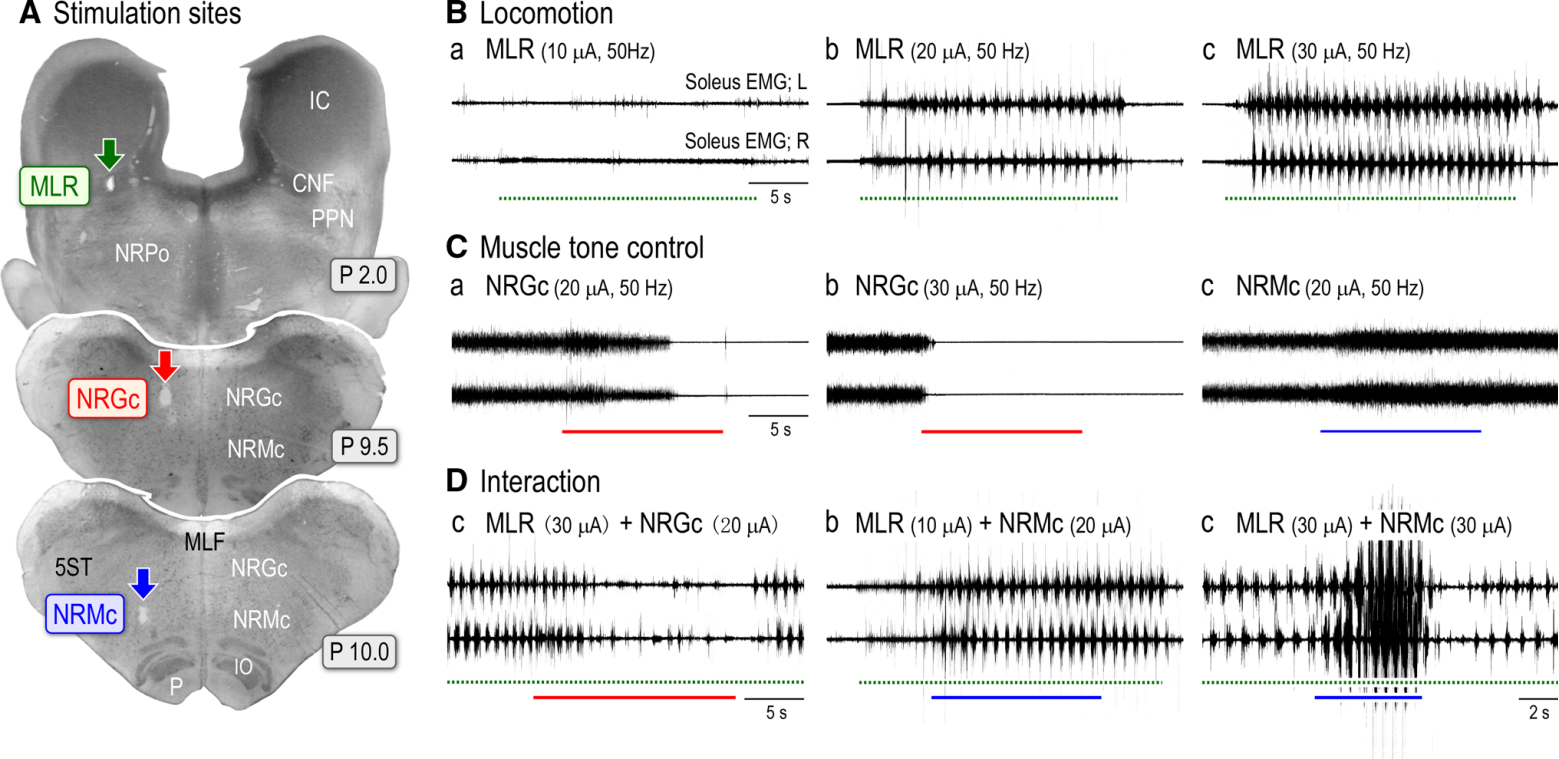
Interaction of the locomotor system and muscle tone control systems. **A** Stimulation sites in the brainstem. MLR (*green arrow*), muscle tone inhibitory site in the NRGc (*red arrow*) and muscle tone increasing site in the NRMc (*blue arrow*). **B** MLR-induced locomotion. Locomotion was not evoked by MLR stimulation with a current of 10 μA (*a*); however, locomotion was evoked by stimulation with current of 20 μA (*b*) and 30 μA (**c**). **C** Muscle tone suppression and augmentation. *a*, *b* Stimulation of the NRGc suppressed postural muscle tone. When the stimulus current was increased from 20 to 30 μA, the latency to atonia was reduced. *c* Stimulation of the NRMc bilaterally increased the level of muscle tone. **D** Interaction of postural muscle tone and locomotion. *a* Stimulation (20 μA) of the NRGc suppressed MLR-induced locomotion. *b* MLR stimulation with a subthreshold current (10 μA) elicited locomotion when combined with NRMc stimulation (20 μA). *c* MLR-induced locomotion was altered from fast walking to a gallop when combined with NRMc stimulation (30 μA). Findings in this figure have not been published previously

**Fig. 9:**
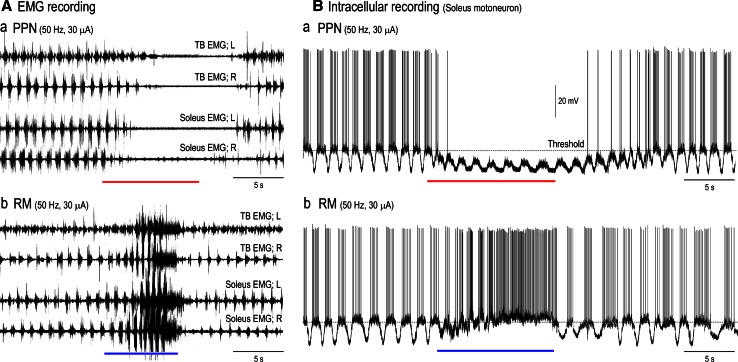
Stimulation of cholinergic and serotonergic nuclei modulates locomotion. **A** *a* In the subthalamic cat preparation, repetitive stimulation applied to the PPN (30 μA, 50 Hz) suppressed locomotion. *b* Stimulation of the RM (30 μA, 50 Hz) initially facilitated locomotion, but subsequently arrested locomotion. EMGs were recorded from both triceps brachii and soleus muscles. **B** Intracellular recording of soleus motoneurons in the same preparation. Motoneurons exhibited rhythmic membrane oscillation and action potentials were generated on the depolarizing phases of the oscillations. *a* PPN stimulation stopped rhythmic firing and hyperpolarized the membrane. Rhythmic membrane oscillations were reduced in size and prolonged in duration during PPN stimulation. *b* Stimulation of the RM initially depolarized the membrane and facilitated rhythmic firing of the soleus motoneuron. Continuation of the stimulation further depolarized the membrane and generated tonic firing of the motoneuron, resulting in suppression of rhythmic oscillations. Rhythmic activity was restored immediately after the RM stimulation was terminated. “Aa” was reported previously (Takakusaki et al. 2003a). Other findings in this figure have not been published

We start at this section by showing core findings in our laboratory. Two lines of evidence are presented. One line elucidates the pontomedullary reticular sites from which microstimulation alters the level of postural muscle tone and locomotion (sections “Effects of electrical stimulation applied to the PMRF”, “Interaction of postural muscle tone and locomotion”, Figs. 6, 7, 8, 9). Another line profiles the characteristics of the medullary RSNs (section “Firing property of the medullary reticulospinal neurons”, Figs. 11, 12, 13, 14).

### Effects of electrical stimulation applied to the PMRF

Effects of microstimulation applied to the PMRF largely alter the activities of the neck, trunk and limb muscles in mesencephalic decerebrate cats. The effects of the reticular stimuli were generally divided into three types; (1) bilateral suppression or inhibition, (2) bilateral augmentation or excitation and (3) non-symmetrical changes in posture such as unilateral limb flexion along with contralateral limb extension (tegmental reflexes).

Sites from which stimulation elicited bilateral suppression and augmentation of soleus EMGs are indicated by red and blue circles, respectively. Bilateral inhibition was evoked by stimuli applied to the dorsomedial part of the PMRF, which corresponded to NRPo (Fig. 6A), the nucleus reticularis pontis caudalis (NRPc; Fig. 6B) and the nucleus reticularis gigantocellularis (NRGc) (Fig. 6C). Inhibitory effects were also elicited by stimuli applied to the dorsolateral mesopontine tegmentum, in an area corresponding to the PPN (Figs. 1, 2, 3, 6A). Muscle tone suppression remained after the stimulation was terminated. Excitatory effects were induced by stimulation that was applied to the dorsal part of the CNF, LC, and ventral part of the PRF at the level of the rostral pons (Fig. 6A). At the cauda pons (Fig. 6B), excitatory sites were located in the ventral part of the NRPc and raphe magnus (RM). In the medulla (Fig. 6C), they were found in the medioventral MRF, in an area corresponding to the nucleus reticularis magnocellularis (NRMc). Following each stimulus, muscle tone augmentation lasted for several seconds or more after the stimulation was terminated.

Tegmental reflexes were induced following stimulation to the sites indicated by open circles in Fig. 6. While this reflex is characterized by extension of the unilateral limb and flexion of the contralateral limb, the direction of such limb movements are opposite to the stimulus applied to the medial and lateral brainstem. Stimulation of the medial sites usually extended the contralateral (right) hindlimb and flexed the ipsilateral (left) hindlimb. These movements were reflected by an increase in the contralateral (right) contraction of soleus muscle and a collapse of ipsilateral (left) soleus muscle contraction. The tegmental reflex evoked from the lateral sites was characterized by extension of the ipsilateral hindlimb and flexion of the contralateral hindlimb. These findings are in agreement with those of previously studies in decerebrate (Sprague and Chambers 1954) and alert (Drew and Rossignol 1990a, b) cat preparations.

Figure 7 summarizes the results from five cats that received reticular stimulation. The findings are superimposed at each level (rostral pons, caudal pons, and medulla) to illustrate the reproducibility of the observed effects. Neural structures in the reticular core were consistently capable of evoking either general inhibitory (red) or general excitatory (blue) effects. The inhibitory sites were invariably located in the dorsomedial part of the PMRF. Stimuli applied to the PPN and its medial part also induced muscular atonia. General excitatory effects were evoked following stimulation of the ventral part of the PMRF in addition to the area that included the LC and RN. While the tegmental reflex (green) was mostly evoked from stimulation to the lateral part of the PMRF, it was also evoked from the medial part of the MPRF if stimuli were applied between the excitatory and inhibitory sites. These findings suggest the presence of functional topography within the reticular core. Specifically general inhibitory and excitatory regions are distributed in the dorsomedial and ventromedial PMRF, respectively. The above findings are line with those reported in previous studies using mesencephalic cat preparations (Habaguchi et al. 2002; Mori et al.^1982^; Oka et al. 1993; Takakusaki et al. 2001). However, the location of the inhibitory sites differs from those reported by Magoun and Rhines (1946) and Lai and Siegel (1988). These authors showed that the general inhibitory effect was evoked by stimuli applied to the ventromedial MRF in decerebrate cat preparations.

### Interaction of postural muscle tone and locomotion

An appropriate level of muscle tone is required to execute locomotor behavior (Mori 1987). Therefore, the locomotor pattern may be altered in accordance with the interaction between the locomotor system and muscle tone control systems. This possibility was experimentally tested (Fig. 8). On the treadmill, stimulation of the MLR (green arrow in Fig. 8A) with 10 μA and 50 Hz did not evoke any movements (Fig. 8Ba). However, increasing the strength of the MLR stimulation (20 μA) elicited locomotion with a cycle time of approximately 1.0 s (Fig. 8Bb). Further increasing the current up to 30 μA facilitated locomotion and the cycle time was reduced to 0.82 s (Fig. 8Bc). In the same cat, stimuli applied to the NRGc (red arrow in Fig. 8A) and the NRMc (a blue arrow in Fig. 8A) abolished (Fig. 8Ca, Cb) and increased (Fig. 8Cc) the level of muscle tone, respectively. Latency to muscular atonia following NRGc stimulation was shortened when the stimulus current was increased from 20 to 30 μA (Fig. 8Ca, Cb). Then, we tested how stimulating the NRGc and NRMc modulated MLR-induced locomotion. Similar to the effect of PPN stimulation in subthalamic cats (Fig. 1Bb), stimulation of the NRGc attenuated MLR-induced locomotion (Fig. 8Da). In contrast, subthreshold stimulation of the MLR (10 μA) elicited locomotion when combined with NRMc stimulation (20 μA; Fig. 8Db). Moreover, MLR-induced locomotion proceeded from fast walking to a gallop if combined with stimulation of the NRMc was increased (Fig. 8Dc).

Records in 9A were obtained from a subthalamic cat preparation. Stimulation of the PPN gradually suppressed spontaneous locomotion along with a reduction in the EMG activity acquired from the forelimbs (triceps brachial muscle; TB) and hindlimbs (soleus muscle; Fig. 9Aa). In contrast, stimulation of the RM facilitated spontaneous locomotion; the locomotor pattern was altered from fast walking to a gallop (Fig. 9Ab). However, continuation of RM stimulation further increased the level of muscle tone, resulting in arrested locomotor rhythm. In this animal, intracellular activity was recorded from hindlimb motoneurons after immobilization (Fig. 9B). Soleus motoneurons exhibited fictive locomotion. Stimulation of the PPN stopped firing and hyperpolarized the membrane. While the oscillations were preserved under the threshold membrane potential, the amplitude was reduced and the duration was prolonged (Fig. 9Ba). This indicates that the activity of the CPG was reduced in addition to decreasing the excitability of the motoneurons. In contrast, stimulation of the RM depolarized the membrane and the oscillations became faster with bursting firings of action potentials (Fig. 9Bb). Continuation of the stimulation further depolarized the membrane and eventually arrested the oscillations. However, the membrane oscillation was immediately restored after termination of the RM stimulation. These results support the previous concept that the appropriate level of postural muscle tone is necessary to elicit locomotion, and that an integration of locomotor signals and excitability of muscle tone control systems is required to produce various patterns of locomotor movements.

The locomotor network in the spinal cord is functionally organized by interneurons that generate locomotor rhythm (i.e., the CPG) and locomotor pattern in addition to motoneurons (Rossignol 1996; Rossignol and Dubuc 1994; Takakusaki 2013). The CPG may consist of interneurons that mediate flexion reflexes. Premotor interneurons, such as Ia interneurons, Ib interneurons and Renshaw cells, may contribute to locomotor pattern formation. Signals from the pattern formation interneurons may be transmitted to the target motoneurons. Supraspinal signals from the cerebral cortex and the brainstem act on these interneurons and motoneurons so that locomotion is initiated and modulated. For example, the muscle tone inhibitory system reduces the excitability of motoneurons in parallel with interneurons in transmission of reflex pathways such as those mediating the flexion reflex, recurrent inhibition (Renshaw inhibition), reciprocal Ia inhibition and Ib inhibition (Takakusaki et al. 2001, 2003b). Suppression of locomotion following the stimulation of the NRGc and PPN may be due to suppression of these spinal interneurons in addition to motoneurons (Figs. 8D, 9A). Consequently, signals from the muscle tone control systems and those from the locomotor system are integrated at the level of the spinal cord.

### Firing property of the medullary reticulospinal neurons

**Fig. 10:**
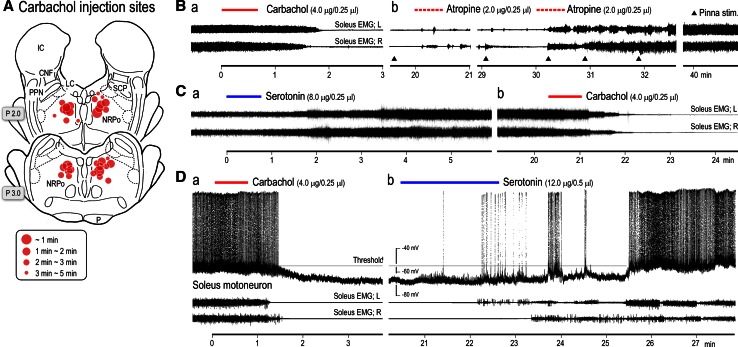
Cholinergic and serotonergic modulation of postural muscle tone. **A** Effective carbachol injection sites in the rostral pons. A carbachol injection was administered in the area where electrical stimulation resulted in collapse of decerebrate rigidity. Sites from which muscular atonia was induced within a short latency are indicated by *large circles*. Muscular atonia was defined as a flatting of the EMG signal acquired from the both soleus muscles. Effective injection sties were located in the dorsomedial part of the medial PRF which corresponded to the NRPo. **B**, **C** EMGs were recorded from both soleus muscles. **B** *a* Muscular atonia induced by an injection of carbachol into the NRPo, and *b* restoration of muscle tone, which was induced by atropine injections into the same site. Pinna stimulation, which was delivered by pinching the scapha (*filled triangles*) aided in restoring the muscle tone. **C** *a* Muscle tone augmentation induced by pontine serotonin injection, and *b* muscular atonia induced by subsequent injection of carbachol into the same site. **D** Simultaneous recording of intracellular activity of a soleus motoneuron and soleus muscle EMGs. A pontine carbachol injection ceased the firing and hyperpolarized the membrane potential of the motoneuron. These changes were associated with disappearance of soleus muscle contraction (**a**). S subsequent pontine serotonin injection restored the excitability of the motoneuron and the level of muscle tone. See the text for further explanation. “A” and “B” are modified from Takakusaki et al. (1993a). “D” is modified from Takakusaki et al. (1993b)

The medial PRF is a major target of cholinergic and serotonergic projections. Cholinergic projections arise from the LDT in addition to the PPN (Lai et al. 1993; Mitani et al. 1988; Semba 1993). Serotonergic projections arise from the raphe dorsalis (RD) and adjacent areas (Semba 1993; Kobayashi et al. 1994). This section begins to show the role of the cholinergic and serotonergic projections to the NRPo in the modulation of the level of postural muscle tone. This was investigated by microinjecting cholinergic and serotonergic agents into the medial PRF, or the NRPo (Fig. 10A), where electrical stimulation elicited muscular atonia in decerebrate cats (Takakusaki et al. 1993a, b, 1994). The location of the carbachol injection sites largely overlapped with the carbachol injection that induces REM sleep in the cat (Baghdoyan et al. 1987; Vanni-Mercier et al. 1989; Yamamoto et al. 1990).

#### Alteration of postural muscle tone following chemical stimulation of the medial PRF

When carbachol (a long-acting cholinergic agonist resistant to choline-esterases) was injected into the right NRPo, postural muscle tone was bilaterally reduced (Fig. 10Ba). Muscle tone was restored by an injection of atropine sulfate into the same site and pinna stimulation (i.e., pinching the scapha indicated by filled triangles; Fig. 10Bb). The carbachol-induced atonia usually lasted for more than 1 h if atropine was not administered. In a different animal, serotonin was injected into the inhibitory site in the NRPo. With this, bilateral contractions of the soleus muscles were increased (Fig. 10Ca). However, the effects were completely suppressed following a subsequent injection of carbachol into the same site (Fig. 10Cb). The carbachol-induced atonia was associated with reduced excitability of the soleus motoneuron (Fig. 10Da). Spontaneous firing of the motoneuron ceased, and the membrane potential was hyperpolarized. The reduced excitability of motoneurons is attributed to both postsynaptic inhibition (Chase and Morales 1990; Takakusaki et al. 1993a) and withdrawal of excitatory inputs impinging on the motoneurons (dis-facilitation; Takakusaki et al. 1993a). Subsequent serotonin injection started to depolarize the membrane and generated action potentials (Fig. 10Db). Several minutes after the injection, firing of the motoneuron was re-established. These changes in the motoneuron were associated with contractions of the soleus muscles. Consequently, the excitability of cholinoceptive and/or serotonin receptive neurons in the medial PRF, specifically the NRPo, is critically involved in the control of postural muscle tone (Takakusaki et al. 1993a, 1994).

#### Firing properties of medullary RSNs in relation to the level of postural muscle tone

**Fig. 11:**
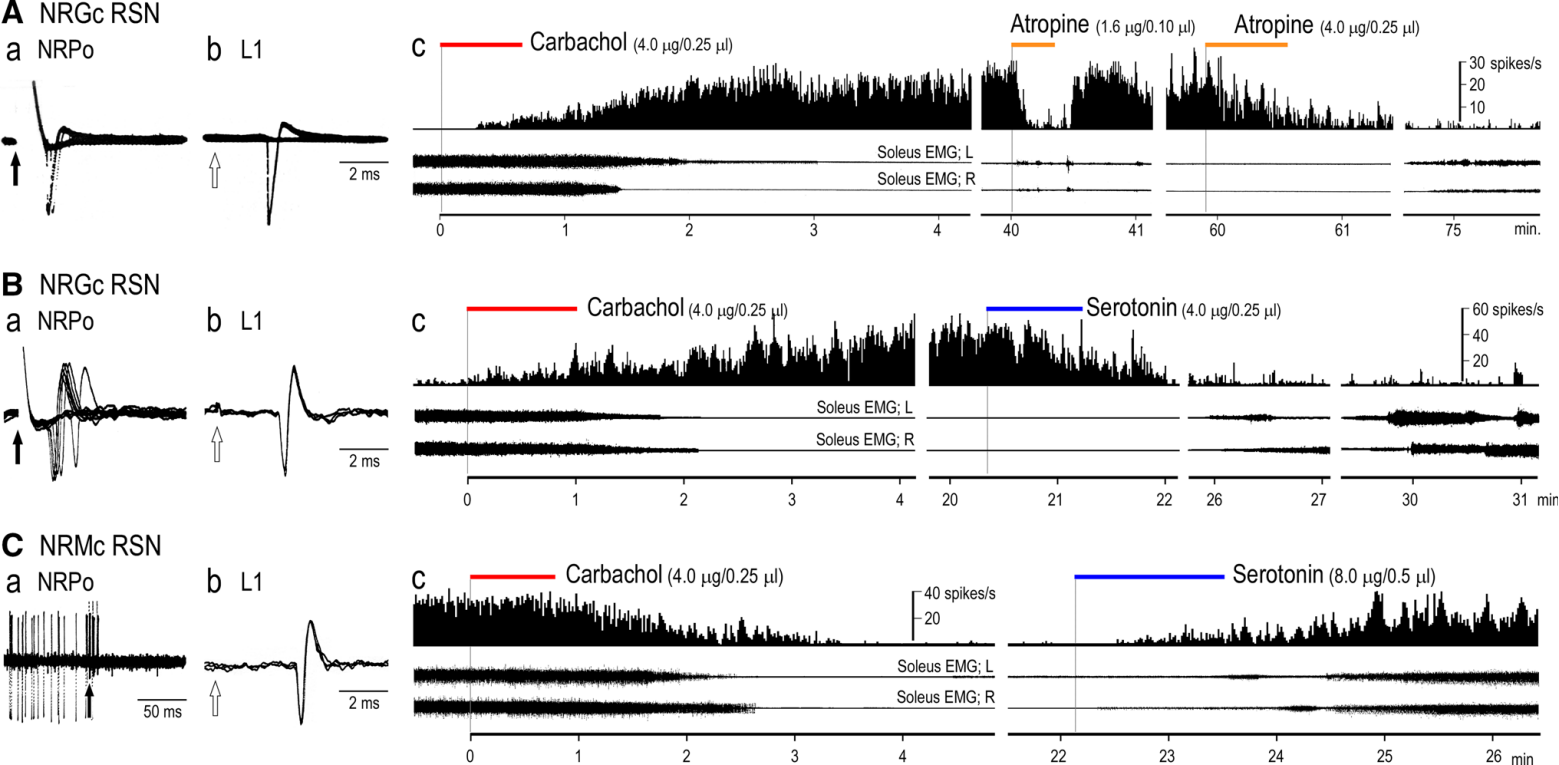
Changes in the activity of medullary reticulospinal neurons (RSNs) induced by pontine injections of carbachol and serotonin. **A**, **B** RSNs in **A** and **B** were recorded from the NRGc. *a* Orthodromic spikes evoked by stimulating the NRPo where repetitive electrical stimulation suppressed postural muscle tone. *b* Antidromic spikes evoked by stimulating the first lumbar segment (L1). The antidromic latency was 2.1 ms for the RSN in **A** (conduction velocity = 103.4 m/s), and 2.5 ms for the RSN in **B** (conduction velocity = 92.4 m/s). *c* The firing frequency of both RSNs was increased by carbachol injections into the NRPo. *d* Subsequent atropine and serotonin injections into the NRPo reduced the firing frequency of the RSN in (**A**) and the RSN in (**B**), respectively. **C** A RSN recorded from the NRMc. *a* Spontaneous firing of the RSN was suppressed by short train pulses of stimuli (three pulses, 5 ms interval, and 30 μA) applied to the NRPo. *b* Antidromic spikes were evoked from the L1 segment with a latency of 3.2 ms (conduction velocity = 69.3 m/s). *c* Spontaneous firing was reduced and then abolished following pontine carbachol injection. *d* A subsequent serotonin injection induced and then increased the firing of the RSN. Findings in “A” and “B” are partly modified from Takakusaki et al. (1994). Findings in “C” have not been published previously

Changes in the firing rates of the medullary RSNs were examined during chemically induced alteration of muscle tone. RSNs in Fig. 11A, B were recorded from the NRGc. They received excitatory input from the NRPo (Fig. 11Aa, Ba) and projected to the lumbar segments (Fig. 11Ab, Bb). Following carbachol injection, the firing frequency of both RSNs was increased in accordance with reduction in muscle tone (Fig. 11Ac, Bc). The RSN in Fig. 11A had a firing frequency of more than 20 Hz and muscular atonia was still evident 40 min after carbachol injection. The firing frequency was reduced by atropine injection; however, the effect was only transient and the firing rebounded after the atropine injection. Approximately 20 min after the first injection, the second higher dose atropine injection reduced the firing frequency of the RSN and partially restored muscle tone. The firing frequency of the RSN in Fig. 11B was greater than 40 Hz 20 min after carbachol injection. In this cat, a subsequent serotonin injection steadily reduced the RSN’s firing frequency. At about 10 min, the firing frequency was less than 10 Hz and the tonic muscle contractions resumed. The RSN in Fig. 11C was recorded from the NRMc. Spontaneous firing of this RSN was suppressed by stimulating the NRPo (Fig. 11Ca), indicating the inhibitory input from the NRPo. Carbachol injection reduced the firing of this RSN as well as muscle tone (Fig. 11Cc). However a subsequent serotonin injection restored the firing frequency of the RSN and the level of muscle tone.

**Fig. 12:**
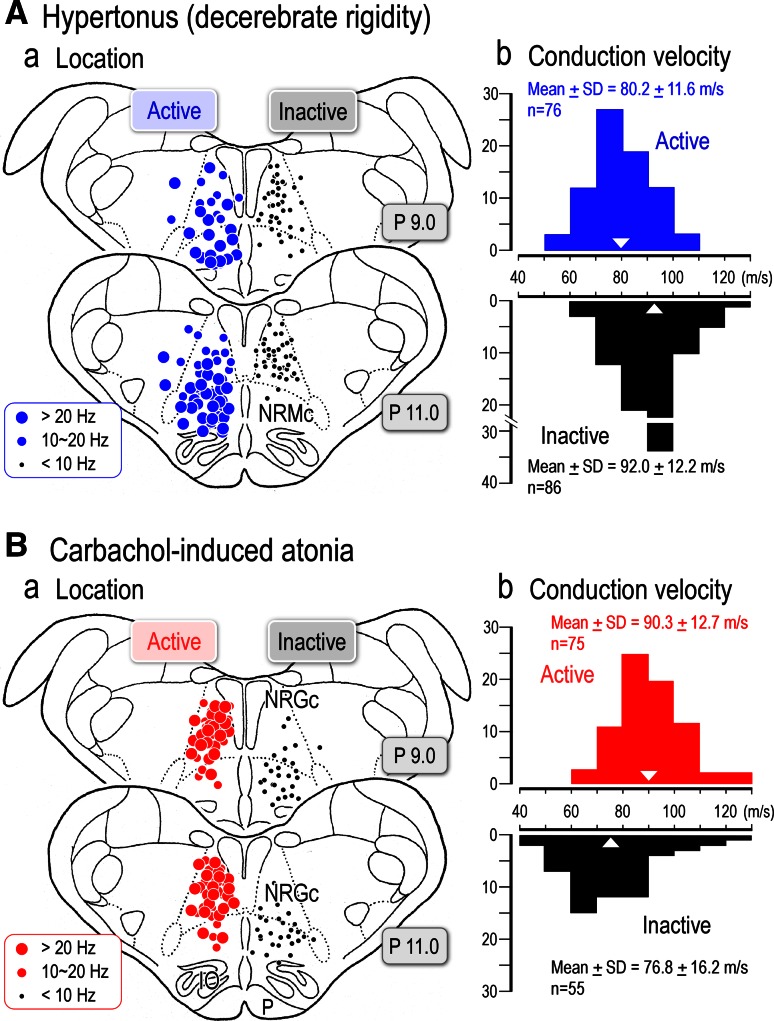
Functional organization of medullary RSNs in relation to the level of muscle tone. **A** *a* Locations of active (*left*) and inactive (*right*) RSNs on the coronal plane of the medulla during hypertonus in decerebrate cats. Active RSNs (*n* = 76) with a frequency higher than 20 Hz (*n* = 52) and those between 10 and 20 Hz (*n* = 24) are indicated by large and small *blue circles*, respectively. Inactive RSNs (*n* = 86) with a firing frequency lower than 10 Hz (most of them were lower than 5 Hz) are indicated by *dots*. *b* Conduction velocity of active (*blue*) and inactive (*black*) RSNs during hypertonus. Active RSNs during the hypertonic state had a slower conduction velocity (mean + standard deviation = 80.2 + 11.6 m/s, *n* = 76) than inactive RSNs (92.0 + 12.2 m/s, *n* = 86). **B** *a* Locations of active (*left*) and inactive (*right*) RSNs on the coronal plane of the medulla during carbachol-induced atonia. The recording was acquired more than 10 min after carbachol injection. Active cells (*n* = 75) with a frequency higher than 20 Hz (*n* = 51) and those between 10 and 20 Hz (*n* = 24) are indicated by large and small *red circles*, respectively. Inactive RSNs (*n* = 55) with a firing frequency lower than 10 Hz are indicated by *dots*. *b* The conduction velocity of active (*red*) and inactive (*black*) RSNs during atonia. Active RSNs during atonia had a faster conduction velocity (90.3 + 12.7 m/s, *n* = 75) than inactive RSNs (76.8 + 16.2 m/s, *n* = 55). The findings in this figure have not been published previously

During the states of hypertonus (before carbachol injection) and carbachol-atonia, the firing frequency, conduction velocity (CV) and the location of medullary RSNs were investigated (Fig. 12).

Here we define RSNs which had firing frequency greater than 10 Hz during hypertonus state and atonia state as hypertonus-related RSNs (*n* = 76 of 162 RSNs) and atonia-related RSNs (*n* = 75 of 130 RSNs), respectively. Hypertonus-related RSNs were located in the ventromedial part of the MRF (blue circles on the left in Fig. 12Aa), whereas atonia-related RSNs were located in the dorsomedial part of the MRF (red circles on the left in Fig. 12Ba). The hypertonus-related RSNs had a CV (80.2 ± 11.6 m/s, mean ± standard deviation; SD, Fig. 12Ab), which was slower than that of the atonia related RSNs (90.3 ± 12.7 m/s, Fig. 12Bb). On the other hand, 86 RSNs were inactive in hypertonus state. These RSNs were mostly distributed in the dorsomedial part of the MRF (denoted by dots on the right in Fig. 12Aa), and had a CV of 92.0 ± 12.2 m/s (Fig. 12Ab). Moreover, inactive RSNs (*n* = 55) during atonia state were located in the ventromedial part of the MRF (Fig. 12Ba). They had a CV of 76.8 ± 16.2 m/s.

#### Properties of medullary RSNs in relation to locomotor control

**Fig. 13:**
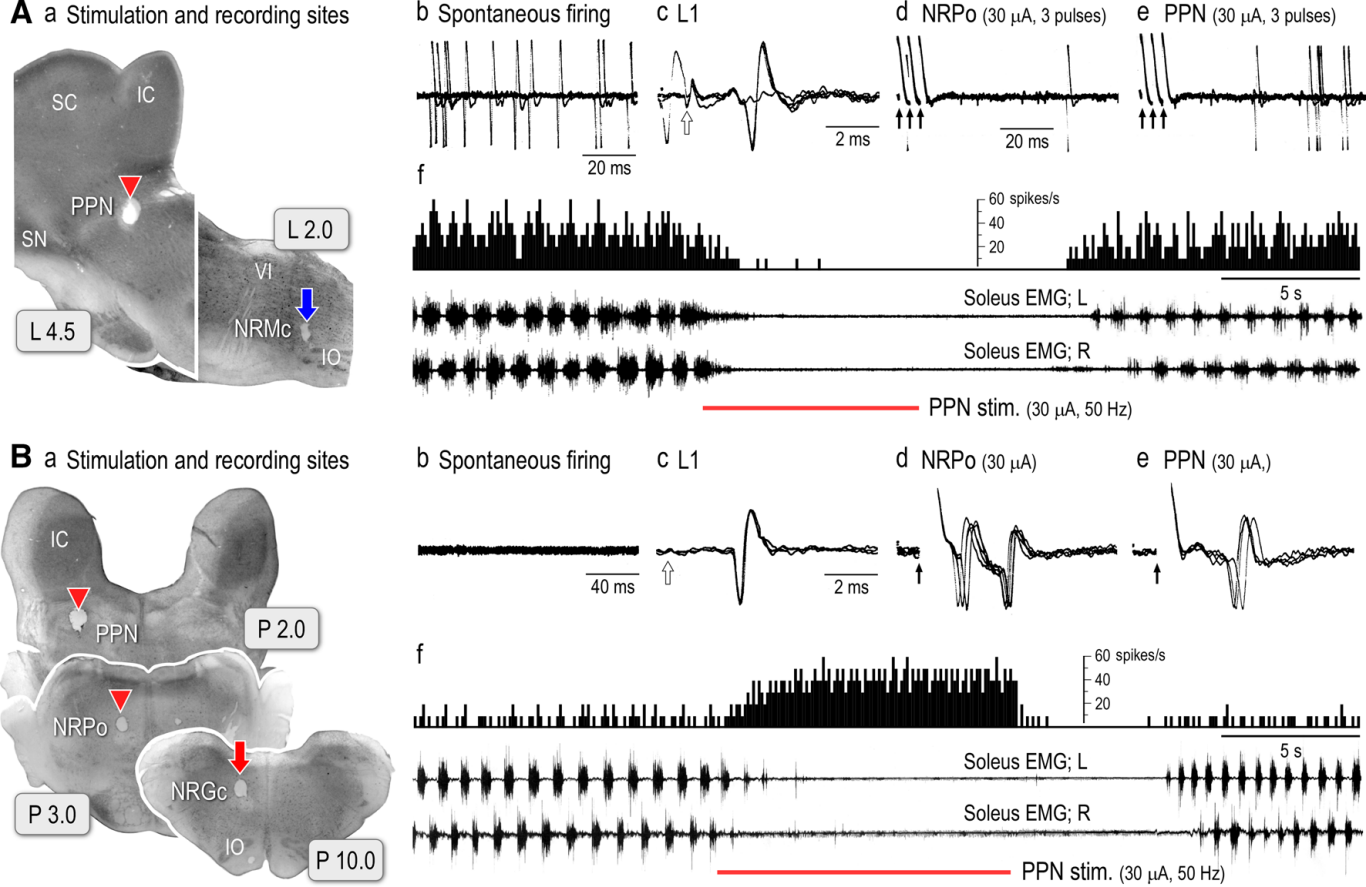
Changes in the firing properties of medullary RSNs during locomotion in a subthalamic cat preparation. **A** *a* A stimulation site in the PPN (*red arrowhead*) and a recording site (*a* *blue arrow*) of a RSN in the NRMc on the parasagittal planes of the lateral and the medial brainstem, respectively. *b* Spontaneous firing during standing. *c* Antidromic spikes evoked by stimulating the first lumbar segment (indicated by an *open arrow*) with a latency of 2.2 ms (conduction velocity = 91.6 m/s). Short train pulses of stimuli (three pulses, 5 ms interval and 30 μA), which is indicted by *upward arrows*, applied to the NRPo (*d*) and the PPN (*e*) blocked the spontaneous firing. *f* During treadmill locomotion, this RSN exhibited rhythmic firing in relation to step cycles. However, the firing and locomotion were completely suppressed following stimulation of the PPN (30 μA, 50 Hz lasting for 8 s). **B** *a* Stimulation sites in the PPN and the NRPo (*red arrowheads*) and a recording site (*a* *red arrow*) of a RSN in the NRGc on the coronal planes of the brainstem. *b* There was no spontaneous firing during standing. *c* Antidromic spikes evoked from the L1 segment (indicated by an *open arrow*) with a latency of 2.4 ms (conduction velocity = 92.0 m/s). Single pulse stimulation (30 μA), which is indicted by *upward arrows*, applied to the NRPo (*d*) and the PPN (*e*) elicited orthodromic spikes with latencies of 1.2 and 2.6 ms, respectively. *f* During treadmill locomotion, this RSN started to discharge with a rate of around 10 Hz. Stimulation of the PPN (30 μA, 50 Hz lasting for 10 s) suppressed locomotion but increased the firing frequency of the RSN. No findings in this figure have been previously published

The firing properties of medullary RSNs have been well characterized during locomotion in acute decerebrate cats (Iwakiri et al. 1995; Orlovsky 1972; Perreault et al. 1993) as well as in chronic alert cats (Drew et al. 1986; Matsuyama and Drew 2000a, b; Schepens et al. 2008). However, the role of medullary RSNs in the integrative control of postural muscle tone and locomotion is unclear. To understand this issue, the firing properties of presumed muscle tone-related medullary RSNs were examined during locomotion. Representative results are shown in Fig. 13, where two RSNs were recorded in high decerebrate (subthalamic) cats. The RSN in Fig. 13A was recorded from the ventromedial MRF (NRMc; Fig. 13Aa). During standing this neuron exhibited spontaneous firing (Fig. 13Ab). This spontaneous firing was inhibited by stimulating the NRPo and the PPN (Fig. 13Ad, Ae), indicating that this NRMc-RSN relates to hypertonus. During locomotion, this RSN exhibited rhythmic firing in relation to the step cycles (Fig. 13Af). However, stimulation of the PPN suppressed the firing of the RSN as well as locomotion (Fig. 13Af). On the other hand, the RSN in Fig. 13B was recorded from the dorsomedial MRF (NRGc; Fig. 13Ba). Because this cell did not exhibit spontaneous firing during standing (Fig. 13Bb), but was orthodromically excited by NRPo and PPN stimulation (Fig. 13Bd, Be), the NRGc-RSN was considered to be an atonia-related RSN. Although the RSN was silent (Fig. 13Bb), it began to discharge with a frequency between 10 and 20 Hz during locomotion (Fig. 13Bf). An activation of spinoreticular tract neurons (Antonino-Green et al. 2002; Huber et al. 1999) during locomotion may induce such a firing of this RSN. The firing frequency was further increased by PPN stimulation, which suppressed locomotion.

**Fig. 14:**
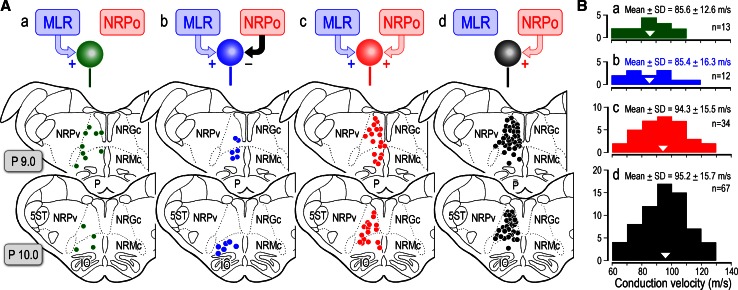
Convergent inputs from the MLR and muscle tone inhibitory region of the medial PRF (NRPo). **A** The location of medullary RSNs in relation to inputs from the MLR and the NRPo. *a* RSNs receiving excitation only from the MLR (*n* = 13). *b* RSNs receiving excitation from the MLR and inhibition from the NRPo (*n* = 12). *c* RSNs receiving excitation from both the MLR and the NRPo (*n* = 34). *d* RSNs receiving excitation only from the NRPo (*n* = 67). **B** The conduction velocity of each group of RSNs. The conduction velocity of RSNs with following NRPo excitation (*c*, *d*) was faster than that without NRPo excitation (*a*, *b*). These data are partly modified from Iwakiri et al. (1995)

Iwakiri et al. (1995) classified medullary RSNs into four groups based on convergent inputs from the muscle tone inhibitory region in the PRF (NRPo) and the MLR. Of 250 RSNs, 126 neurons were excited by stimulating either the MLR (*n* = 25) or the NRPo (*n* = 67) or both (*n* = 34), indicating that half of the medullary RSNs contributed to the control of muscle tone and locomotion. Group 1 RSNs (*n* = 13) was excited following stimulation only from the MLR (13/59 = 22.0 %, Fig. 14Aa). Group 2 RSNs (*n* = 12) was excited following stimulation to the MLR and inhibited following stimulation to the NRPo (Fig. 14Ab). Group 3 RSNs (*n* = 34) was excited following stimulation to both the MLR and the NRPo (Fig. 14Ac). It should be noted that the majority (46/59 = 80.0 %) of MLR-activated RSNs received either inhibitory (Group 2 RSNs; 12/59 = 20.3 %) or excitatory (Group 3 RSNs; 34/59 = 57.7 %) inputs from the NRPo. Group 4 RSNs (*n* = 67) were excited following stimulation to the NRPo (Fig. 14Ad). We found that the conduction velocity was faster in RSNs that belonged to the Group 3 (94.3 ± 15.5 m/s) and Group 4 (95.2 ± 15.7 m/s) than for those in the Group 1 (85.6 ± 12.6 m/s) and Group 2 (85.4 ± 16.3 m/s; Fig. 12B).

We compared the locations of RSNs in relation to the control of postural muscle tone (Fig. 12) and locomotion (Fig. 14). In the Groups 1 and 2, the majority of the RSNs (18/25; 75 %) were distributed in the ventral part of the MRF corresponding to the NRMc (Fig. 14Aa, Ab). This distribution was similar to that reported by Garcia-Rill and Skinner (1987) who demonstrated that stimulation of the MLR mostly activated RSNs located in the ventromedial medulla. Moreover, the distribution is in agreement with the location of that of hypertonus-related RSNs (Fig. 12A) suggesting that these MLR-activated RSNs also contribute to increasing the level of muscle tone. The majority of RSNs in the Groups 3 and 4 (Fig. 14Ac, Ad) was located in the dorsomedial MRF corresponding to the NRGc, and their distribution was similar to that of the atonia-related RSNs (Fig. 12B). It is possible that the Group 3 RSNs (*n* = 34) was involved in muscle tone suppression and locomotor control. Surprisingly, only 13 RSNs (22.0 %) were excited following stimulation from the MLR without the influences of the NRPo (Group 1). In other words, considerable portion of the medullary RSNs may integrate supra-medullary signals related to the control of postural muscle tone and locomotion. Descending signals in these RSNs thus modulate the activity of the spinal locomotor network such that the level of muscle tone and locomotor patterns are simultaneously regulated.

## Functional organization of the PMRF

### Mechanisms involved in the alteration of functional organization of the PMRF

The lines of evidence described above in decerebrate cat preparations suggest the presence of a gross functional topography of the reticulospinal systems; this functional topography likely contributes to the control of postural muscle tone. Specifically, RSNs in the dorsomedial MRF are involved in inducing muscular atonia (i.e., a general inhibitory effect). Conversely, RSNs in the ventromedial MRF may be involved in muscle tone augmentation (i.e., a general excitatory effect).

Magoun and Rhines (1946) were the first to demonstrate that the PMRF exerted either a general excitatory or inhibitory influence on motoneurons at all levels of the neuraxis in decerebrate cats. Since then the PMRF has been viewed as a structure that does not have a distinct differentiation of its cellular features (Brodal 1981; Scheibel and Scheibel 1958) or function (Siegel 1979). Subsequent studies, however, show that the functional organization of the PMRF is conceptually different. This difference can be caused by differences in the experimental animal models employed. In the anesthetized cat preparation, Peterson and his colleagues show that the PMRF is organized with sufficient specificity to mediate certain types of discrete motor acts (Peterson 1984; Peterson et al. 1978, 1979). In the chronic alert cat preparation, the organization of the PMRF, on the other hand, functions to control coordinated patterns of movement rather than the production of discrete individual limb movements (Drew and Rossignol 1990a, b). Moreover, the effects of reticular stimuli are state-dependent (Chase and Wills 1979; Wills and Chase 1979; Chase et al. 1986; Lai et al. 2010). Specifically, reticular stimulation elicits excitatory effects during wakefulness, whereas identical stimuli invariably produce inhibitory effects during REM sleep. This phenomenon is called state-dependent response reversal. Chase (1980) suggests that the motor functions of the reticular formation are multifaceted and state-determined.

This led to a critical question; how is a particular reticular formation motor function selected from its various and multifaceted functions depending on the behavioral states in animals? The activities of the cholinergic and monoaminergic (serotonergic and noradrenergic) neurons are reciprocally regulated depending on the sleep-awake cycles (Datta 2002; Koyama and Sakai 2000; Koyama et al. 1994; Kubin 2001, Pace-Schott and Hobson 2002; Sakai and Crochet 2004; Trulson et al. 1981). During wakefulness, the activity of monoaminergic neurons is greater than that of cholinergic neurons, while both groups of neurons are active. In contrast, the firing frequency of cholinergic neurons is much higher than that of monoaminergic neurons during REM sleep. Therefore, medial PRF (NRPo) neurons may exhibit excitability modification in a state-dependent manner, as they receive cholinergic and monoaminergic inputs. Medial PRF neurons with excitatory ACh and inhibitory serotonin responses, which were shown by Greene and Carpenter (1985), may trigger muscle tone suppression. Conversely, PRF neurons that exhibit inhibitory ACh and excitatory serotonin responses may be involved in muscle tone augmentation. Xi et al. (2004) suggested that the cholinergic control of the PRF neurons is gated by local GABAergic neurons; the cholinoceptive PRF neurons may lead REM sleep when the GABAergic neurons are inactive, whereas an activation of the GABAergic neurons may result in the generation of wakefulness. However, involvement of serotonin in this gating mechanism is not certain.

Such transmitter-specific and state-dependent excitability modification of PRF neurons may preferentially excite either the atonia-related or the hypertonus-related medullary RSNs (Fig. 12). Then, the RSNs that exhibit preferential excitability would be recruited to behave in their preferred manner (i.e., neurons that are preferentially excitable would exhibit excitation and vice versa), as suggested by Edelman (1987). Because these two groups of RSNs are intermingled within the medial MRF, the effects of reticular stimuli during REM sleep may induce muscular atonia when the excitability of atonia-related RSNs is higher than that of hypertonus-related RSNs. In contrast, reticular stimuli during wakefulness (i.e., the alert state) may preferentially activate hypertonus-related RSNs so that reticular stimuli may produce coordinated patterns of movements together with certain types of discrete motor acts in addition to increasing the level of muscle tone.

Based on the abovementioned considerations, the following proposition can be made. The PMRF is functionally organized not only as a composition of specific regions that evoke particular patterns of movements, but also as a homogeneous or non-specific region from which generalized motor inhibition is produced. The non-specific organization of the PMRF may be involved in the generalized motor inhibition that occurs during REM sleep. Changes in the functional organization of the PMRF may therefore be crucial for the expression of state-dependent motor behaviors.

### Organization of reticulospinal systems in the control of postural muscle tone and locomotion

**Fig. 15:**
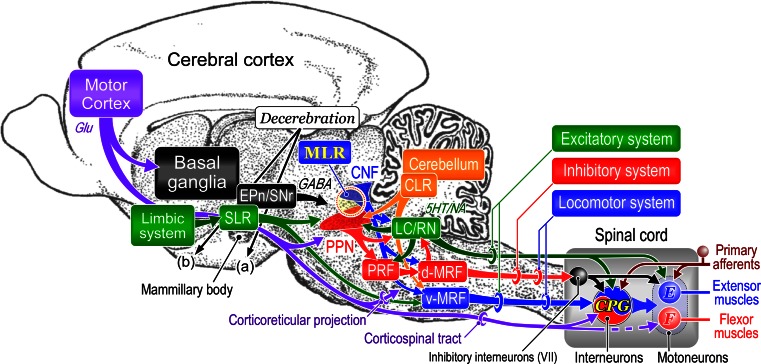
Basic signal flow involved in the control of muscle tone and locomotion in the cat. MLR may include the CNF and dorsal part of the PPN. Optimal sites for evoking locomotion were located in the ventral part of the CNF ("Functional organization of the lateral part of the mesopontine tegmentum"). However, the effects of the mesopontine stimuli may largely depend on the excitability of the PPF neurons ("Consideration of experimental procedures and limitation of approach"). Muscle tone inhibitory system is considered to arise from the cholinergic neurons in the PPN, which may sequentially activate PRF neurons and RSNs in the dorsal part of the MRF (d-MRF). This inhibitory system possibly activates inhibitory interneurons, which inhibit both the CPG and motoneurons so that locomotor movements as well as postural muscle tone are suppressed ("The inhibitory system"). Monoaminergic descending pathways arising from the LC and RN are considered as muscle tone excitatory system. The reticulospinal system descending from the ventral MRF may also contribute to the augmentation of muscle tone ("The excitatory system"). The level of postural muscle tone can be regulated by the interaction between the inhibitory and excitatory system ("Interaction of postural muscle tone and locomotion, The inhibitory system"). There is interconnection between the PPN and the RN. The PRF is one of important site where both serotonergic and cholinergic neurons projected and regulate the level of postural muscle tone by modulating the muscle tone control systems. Signals from the MLR (both the CNF and PPN) may recruit RSNs, which belong to the excitatory and inhibitory systems, and activate CPGs in the spinal cord to elicit locomotion ("The locomotor system"). Locomotor pattern can be altered by the balance between the muscle tone control systems and locomotor system "Interaction of postural muscle tone and locomotion, Properties of medullary RSNs in relation to locomotor control"). Signals from the limbic system may control locomotion via the SLR, which activate the RSNs in the ventral MRF via direct or indirect projection via the MLR ("Efferents from the limbic-hypothalamic systems"). The basal ganglia have direct projections to the mesopontine tegmentum and control postural muscle tone and locomotion ("Efferents from the basal ganglia"). Similarly, motor cortical areas have projections to the mesopontine tegmentum and PMRF (cortico-reticular projection) in addition to the corticospinal tract ("Efferents from the cerebral cortex"). CLR, which corresponds to the connecting fibers between bilateral fastigial nuclei, may mostly control locomotion via projections to the PPN and to the PMRF ("Efferents from the cerebellum"). **a** A level of decerebration at the precollicular-postmammillary level (the mesencephalic cat preparation). **b** A level of decerebration at the precollicular-premammillary level (the subthalamic cat preparation)

Followings are our current understanding as to the organization of descending systems involved in the control of postural muscle tone and locomotion based on our findings in addition to results obtained from other laboratories. The organization of these systems is schematically illustrated in Fig. 15.

#### The inhibitory system

A series of studies in our laboratory suggested that the muscle tone inhibitory system arises from cholinergic neurons in the PPN (Takakusaki et al. 1993a, 1994, 2001, 2003a, 2004a, c, 2005, 2011). Although the PPN is composed of a heterogeneous neuronal population, cholinergic neurons may activate cholinoceptive neurons in the medial PRF (NRPo), which in turn excite medullary RSNs located in the dorsomedial MRF (atonia-related RSNs; Fig. 12B). This inhibitory system inhibits α- and γ-motoneurons that innervate extensor and flexor muscles, in parallel with interneurons mediating reflex pathways via a group of spinal inhibitory interneurons in the Rexed lamina VII (Takakusaki et al. 2003b). Presynaptic inhibition of primary sensory afferents was also induced by this system (Chan and Barnes 1974; Takakusaki 2015). Accordingly, an activation of this system may inhibit whole neuronal constituents of spinal reflex loops including the CPG, resulting in the suppression of locomotion as well.

However, critical questions remain unanswered. First, the medullary-induced inhibitory effects are presumably mediated by neurons with a CV of 20–40 m/s (Habaguchi et al. 2002; Kohyama et al. 1998), which is much slower than that of atonia-related RSNs (Fig. 12Bb). The second question involves the lamina VII interneurons. Postsynaptic inhibition uses glycine and presynaptic inhibition uses GABA; therefore there is a need to determine whether the lamina VII interneurons contain both transmitters and contribute to both inhibitory processes. It should be noted that this muscle tone inhibitory system likely induces muscular atonia during REM sleep (Chase and Morales 1990). Similarly, cholinergic projection from the PPN to the paramedian MRF in the caudal medulla is proposed to induce atonia during REM sleep (Shiromani et al. 1990).

#### The excitatory system

Muscle tone augmentation was induced by applying stimulation to the monoaminergic nuclei in the rostral pons such as the LC and RD, the RM in the caudal pons, and the raphe pallidus (RPa) in the medulla (Figs. 3, 6). These findings support a previous suggestion that descending monoaminergic pathways such as the coerulospinal and raphespinal tracts may comprise the muscle tone excitatory system (Fung and Barnes 1981; Holstege and Kuypers 1987; Sakai et al. 2000). Moreover, the reticulospinal tract descending from the ventromedial MRF may also belong to the muscle tone excitatory system (Figs. 6, 7, 8, 12). Because a population of reticulospinal fibers adjoining the raphe nuclei contains serotonin (Holstege and Kuypers 1987), the hypertonus-related medullary RSNs might use serotonin to increase the level of muscle tone. Consequently, serotonergic projection systems may play a vital role in modulating the level of muscle tone by direct projections to the spinal cord and indirect projections via the medullary reticulospinal tract. It is also possible serotonergic projections to the PRF and PPN may reduce the excitability of the inhibitory system (see also section “Properties of medullary RSNs in relation to locomotor control”, Fig. 15).

During muscle tone augmentation induced by pontine serotonin injection (Fig. 10), postsynaptic excitation and presynaptic facilitatory effects are induced in motoneurons (Takakusaki et al. 1993b). Such excitatory synaptic mechanisms may be induced by the activation of the ventromedial medullary RSNs (Fig. 12A). Alternatively these facilitatory effects are possibly attributed to the removal of inhibitory effects that are mediated by the atonia-related medullary RSNs (Fig. 12B).

#### Interaction between the inhibitory and excitatory systems

Postural muscle tone is regulated by a counterbalance between the excitatory and inhibitory systems (Fig. 15). Such interactions may occur at the levels of the brainstem and spinal cord as follows. (1) Reciprocal connection exists between the cholinergic and monoaminergic nuclei. The mesopontine cholinergic neurons receive direct serotonergic projection from the DR and noradrenergic projection from the LC (Honda and Semba 1994). These monoaminergic projections may inhibit cholinergic neurons (Kobayashi et al. 2003; Leonard and Llinás 1994; Pal and Mallick 2006). Conversely, cholinergic input to DR inhibits serotonergic neurons by the mediation of GABA neurons (Yang and Brown 2014). (2) The medial PRF (NRPo) receives monoaminergic projections from the DR/LC (Semba 1993) and cholinergic projections from the PPN/LDT (Mitani et al. 1988; Lai et al. 1993). Therefore, the activity of each muscle tone control system is determined by the excitability of NRPo neurons in response to ACh and serotonin. (3) MRF neurons belonging to the inhibitory system may suppress the activity of LC neurons (Mileykovskiy et al. 2000). (4) Reciprocal changes in the muscle tone control systems are reflected by the release of neurotransmitters in the spinal cord. For example, Lai et al. (2010) demonstrated a combination of increased release of glycine and GABA and decreased release of serotonin and noradrenalin during atonia following stimulation of the medial MRF.

#### The locomotor system

Previous studies suggested that signals from the MLR activate medullary RSNs which comprise the “locomotor system” that relays locomotor commands to the spinal locomotor network so that it generates an oscillatory pattern of locomotion (Grillner 1981; Armstrong 1986; Garcia-Rill and Skinner 1987; Rossignol 1996). Presumably, the RSNs in the locomotor system utilize glutamate to elicit the locomotor rhythm (Fenaux et al. 1991; Douglas et al. 1993; Hagevik and McClellan 1994; Jordan et al. 2008; Rossignol and Dubuc 1994). As shown in Figs. 12, 13, and 14, RSNs constituting the locomotor system may include a considerable population of the medullary RSNs which relate to the control of muscle tone, indicating that the level of muscle tone and the locomotor rhythm are simultaneously regulated by these RSNs (Fig. 15). MLR-induced locomotion is often preceded by the augmentation of postural muscle tone (Fig. 1), which is reflected by membrane depolarization of extensor motoneuron (Fig. 4). The muscle tone augmentation can also be ascribed to the recruitment of the monoaminergic muscle tone excitatory system, such as the coerulospinal and raphespinal tracts (Mori et al. 1992). The raphespinal tract is particularly important in the control of posture and locomotion (Jacob and Fornal 1993; Sławińska et al. 2012, 2014). Both the firing rates of serotonin neurons in the caudal RN (Veasey et al. 1995) and the serotonin levels in the spinal cord increase during locomotion (Gerin and Privat 1998). Xiang et al. (2013) used pseudorabies virus, which is a marker for synaptic connectivity in CNS by propagating retrogradely through chains of functionally connected neurons, and revealed that neurons in the area corresponding to the MLR were retrogradely infected via the medullary RSNs in mice. However, no serotonergic and catecholaminergic neurons were infected, indicating without the participation of the monoaminergic pathways in the MLR-induced locomotion.

## Forebrain and cerebellar control of the reticulospinal system

Forebrain structure including the cerebral cortex, the limbic-hypothalamic structures, and the basal ganglia as well as the cerebellum control posture and gait largely by acting on the reticulospinal system through their direct and indirect connections via the MLR (Fig. 15). These cortical and subcortical projections may enable animals to express volitional and emotional motor behaviors depending on the context (Grillner et al. 1997; Takakusaki 2008). In this section, we consider how signals from these structures modulate the excitability of the mesencephalic and PMRF in order to assist understanding of the mechanisms of controlling posture and locomotion and interpretation of the pathophysiological bases underlying the posture-gait disturbances in various neurological disorders.

### Efferents from the limbic-hypothalamic systems

Regardless of the nature of emotional stimuli, they usually elicit responses related to alertness that produce stereotyped movements such as increased postural muscle tone or locomotion, which accompany autonomic sympathetic responses (Swanson and Mogenson 1981). Projections from the limbic-hypothalamic systems to the brainstem via the medial forebrain bundle may play crucial roles in these processes. Stimulation of different parts of the hypothalamic areas elicited various types of goal-directed behaviors (Sinnamon 1993). In cats with chronically implanted electrodes, stimulation of the SLR elicited alerting responses followed by exploratory (searching) or defensive behaviors (Mori et al. 1989). Stimulation of the SLR evoked locomotion after large lesion was made in the MLR area (Shik and Orlovsky 1976). Therefore, the SLR has a direct projection to the ventral MRF area and thus activates the locomotor system (Sinnamon and Stopford 1987). On the other hand, stimulation of the MLR abruptly elicited machine-like explosive locomotion (Mori et al. 1989). Three types of emotional motor systems that function in different behavioral or motivational contexts have been proposed; (1) an appetitive system, (2) a primary defensive system and (3) an exploratory system. All eventually converge on the mesopontine tegmentum. The appetitive system is composed of the preoptic and perifornical lateral hypothalamic area, which mostly corresponds to the SLR, and their connections with the brainstem. Orexin neurons in the perifornical lateral hypothalamic area may contribute to this system (Okumura and Takakusaki 2008; Sakurai 2007). The primary defensive system consists of the perifornical and medial hypothalamic area, plus their connections with the central gray and the CNF. The exploratory system may involve the subpallidal area, including the nucleus accumbens, zona incerta and PPN (Grillner et al. 1997). The CNF and PPN may, therefore, include subcomponents involved in emotional locomotor behaviors under different contexts.

In narcoleptic patients, however, emotional signals elicit a sudden loss of muscle tone (cataplexy; Nishino 2003). How do emotional stimuli elicit atonia instead of locomotion in narcolepsy? Postmortem studies revealed that orexin neurons in the perifornical lateral hypothalamus are largely damaged in narcolepsy (Thannickal et al. 2000). Orexin neurons are active during wakefulness but inactive during REM sleep (Koyama et al. 2003; Mileykovskiy et al. 2005). Orexin neurons have dense projections to the mesopontine tegmentum; these neurons also project to brainstem aminergic nuclei and most other brain areas (Peyron et al. 1998). Therefore, orexinergic projections to the mesopontine tegmentum may activate the locomotor system and muscle tone excitatory system but suppress muscle tone inhibitory system (Takakusaki et al. 2005). Consequently, in the presence of orexin (normal awake state), emotional signals reaching the midbrain may increase the level of muscle tone so that locomotor behaviors can be elicited. However, in the absence of orexin, excitability of the inhibitory system would be greater than that of the locomotor and excitatory systems; emotional signals may preferentially activate the inhibitory system, resulting in muscular atonia. We propose that the emotional motor system from the limbic system to the midbrain contribute to the pathophysiological mechanisms of cataplexy in narcolepsy.

### Efferents from the basal ganglia

Garcia-Rill et al. (1983a, b, 1986) first suggested that GABAergic efferents from the basal ganglia to the mesopontine tegmental area provided a clear and straightforward view on the locomotor function of the basal ganglia. The mesopontine tegmentum including the PPN receives substantial input from the basal ganglia, particularly the substantia nigra pars reticulata (SNr) (Beckstead et al. 1979; Saitoh et al. 2003, Spann and Grofova 1991). Subsequent studies in rats, cats, and monkeys suggest the existence of the descending basal ganglia projection system to the spinal cord via the brainstem structures. In the rat, Sherman et al. (2015) showed that a group of glutamatergic spinally projecting neurons in the lateral pontine tegmentum received GABAergic projection from the SNr. The authors suggest that dysfunction of these neurons may relate to cataplexy in narcolepsy patients in addition to posture-gait disturbances in PD. On the other hand, the SNr, specifically dorsolateral part of the SNr has direct ipsilateral projection to the lateral part of the PMRF of the rat (Yasui et al. 1995, 1997). However, there was no direct evidence of the contribution of the direct SNr-PMRF in the control of posture and locomotion. In the cat using electron microscopy, Nakamura et al. (1989) revealed the projection from the SNr to the medial MRF via neurons in the PPN. In decerebrate cat preparation, Takakusaki et al. (2003a, 2004a, 2005, 2011) further demonstrated that MLR (CNF) and the inhibitory region in the PPN receive GABAergic projection from the SNr. Moreover, efferents from the SNr may use different channels to control posture and locomotion; efferents from the lateral part of the SNr to the PPN modulate postural muscle tone, whereas those from the medial part of the SNr to the MLR (CNF) modulate locomotion. In the monkey, Rolland et al. (2011) demonstrated the presence of pallidal and nigral projections, possibly GABAergic, to the neurons in the PPN and CNF, which in turn have projections to the PMRF. Specifically, the authors hypothesized that the nigral-CNF pathway controls axial posture, whereas the pallidal-PPN pathway modulate locomotion.

Important issues remain to be solved, however. First, it is still unknown whether the same mechanisms exist in humans. Functional imaging studies during mental imagery indicated subcortical structures involvement in postural and locomotor control in humans (Jahn et al. 2008a, b), with the most important regions being the MLR, SLR and cerebellar locomotor region (CLR), in addition to the PMRF. Second, it is unclear what drives or dictates the SNr-induced control of locomotion and posture. There are sub-compartments in the basal ganglia: neostriatum-dorsal pallidal pathway and the ventral striatum-ventral pallidal pathway (Lynd-Balta and Haber 1994). Mogenson and collaborators (Brudzynski et al. 1993; Mogenson 1991; Slawinska and Kasicki 1995; Swanson and Mogenson 1981) showed that GABAergic projections from the nucleus accumbens to the MLR via the ventral pallidum participate in locomotor control. The nucleus accumbens receives dopaminergic inputs from the ventral tegmental area and conveys reward-related motivational signals in rodents (Nicola et al. 2004), monkeys (Schultz 2013), and humans (Knutson et al. 2003; Pagnoni et al. 2002). Because it also receives inputs from the hippocampus and amygdala, the ventral pathway may be involved in reward-oriented locomotor behaviors. On the other hand, the more recently evolved parts of the basal ganglia make up the dorsal system, which consists of the neostriatum (caudate nucleus and putamen) -dorsal pallidum (globus pallidus and substantia nigra reticulata; GP-SNr). This system may exert locomotor control depending on the cognitive/behavioral context, such as sensory-guided locomotor control.

Because basal ganglia output is considered to be increased in patients in PD (DeLong and Wichmann 2007; Filion and Tremblay 1991; Nambu 2008), it is reasonable to consider that excessive GABAergic inhibitory effects on neurons in the mesopontine tegmentum, specifically, on the remaining cholinergic and non-cholinergic neurons, may be one of the pathophysiological bases of posture and gait disturbances in this disease (Nutt et al. 2011; Takakusaki et al. 2004c, 2008, 2013). Similarly, as suggested by Müller et al. (2013), reduced activities in the cholinergic PPN neurons may also disturb multi-sensory integration at the level of the thalamus to produce postural impairments (Müller et al. 2013, section “Cholinergic systems participating in the gait control via their role in modulating attention”). Based on these considerations, it is sufficiently significant to investigate whether the impaired motor functions are restored by reducing the suppressive effects from the SNr. This was clinically examined by Chastan et al. (2009) who demonstrated that high frequency (130–190 Hz) DBS applied to the SNr improved axial motor symptoms, such as gait failure and postural disturbances, in patients with PD. Possibly, the firing of the SNr neurons cannot respond to the high-frequency stimulation due to depolarization block (Takakusaki et al. 2003a, 2011).

### Efferents from the cerebral cortex

When a locomoting subject encounters obstacles, each foot must be placed with a high degree of accuracy. This accuracy requires precise visuomotor coordination through the visuo-parieto-frontal cortical projection (Marigold and Drew 2011), as in the subject has to modify the limb trajectory in each step to achieve appropriate foot placement (Georgopoulos and Grillner 1989). When pyramidal tracts were bilaterally transected in cats, basic locomotor synergy was generally not disturbed (see Armstrong 1986). Deficits in simple locomotion were surprisingly negligible and the most important defect was hyperextension of the hindlimb during the stance phase (Eidelberg and Yu 1981); however, skilled locomotor tasks such as precise foot placement were severely impaired. Lesions in the motor cortices elicited the same qualitative effects as the pyramidal tract transection (Liddle and Phillips 1944). Skilled performance was more severely disturbed by postcruciate than by precruciate lesions. After postcruciate lesions including both the somatosensory cortex and the parietal cortex, the cat refused to walk on narrow tracks (postcruciate syndrome; Adkins et al. 1971). The precruciate area, which corresponds to the supplementary motor area (SMA) and the premotor area (PM) in primates, may be involved in movement initiation; the postcruciate cortices may utilize specific somatosensory inputs to fulfill a role in the regulation of ongoing movements (Brooks and Storney 1971) via anticipatory or feed-forward adjustments (Massion 1992; Vicaro et al. 1983).

In non-human primate, Nakajima et al. (2003) demonstrated that muscimol (GABA_A_ agonist) injections that inactivated the leg region of the M1 resulted in paresis of the contralateral leg engaged in unrestrained bipedal walking in monkey. On the other hand, muscimol injections into the trunk/leg regions of the bilateral SMA largely disturbed postural control without inducing paralysis (Mori et al. 2003). When an injection was administered into the dorsal PM, spontaneous walking was maintained in the monkey; however, the monkey could not start locomotion using sensory cue. These findings indicate the presence of functional organization in the motor cortical areas in the control of posture and locomotion in bipedal walking animals. Then, how do cortical outputs control posture and movements? Studies using neural tracers showed abundant cortico-fugal projections to the brainstem reticular formation from the premotor cortices in monkey (Keizer and Kuypers 1989) as well as quadruped animals (Matsuyama and Drew 1997). Matsumura et al. (2000) showed that the projection from the primary motor cortex (M1) to the PPN had a gloss somatotopical representation: orofacial, forelimb, and hindlimb representations tended to be arranged in orderly manner from medial to lateral. Moreover, inputs from the M1, SMA and PM largely overlapped. They concluded that the PPN received partially separate but essentially convergent cortical inputs not only from multiple motor-related areas representing the same body part, but also from multiple regions representing diverse body parts. Similarly, using probabilistic diffusion tractography in a rhesus monkey, Aziz and his colleagues (Aravamuthan et al. 2009) demonstrated that the SMA and the dorsal PM had strong connections with the lateral and medial PPN, respectively. Evidence suggests that the SMA contributes to anticipatory postural adjustment for step initiation, and this process is impaired in patients with Parkinson’s disease (Jacobs et al. 2009). It has been suggested that the projection from the cerebral cortex to the vermis is also part of the neural substrate for anticipatory postural adjustments and dysfunction of this system may underlie some forms of dystonia (Coffman et al. 2011).

In human, Fling et al. (2014) used functional neuroimaging approach and revealed strong functional connectivity between the SMA and MLR area, which was positively correlated with freezing severity in patients of PD. In contrast, connectivity between the STN and SMA was lost. They suggested that the former connectivity may potentially due to a maladaptive compensation, and the latter may reflect the reduced automatic control of gait by the basal ganglia. Karachi et al. (2012) propose two distinct networks between cerebral cortex and the lateral mesencephalon: one involving motor/premotor cortices and the cerebellum (motor loop) with the participation of the CNF and dorsal PPN for the walking task and the other involving posterior parietal and dorsolateral prefrontal cortices with the participation of the ventral PPN (cognitive loop) for the object moving task. They suggested that the involvement of cortico-mesencephalic projection contribute to different aspects of gait control: the former controls motor aspects of locomotion and the ventral PPN being involved in integrating sensory information. In addition, functional connections of these loops with the subcortical locomotor networks are particular important with respect to aging. The supraspinal locomotor centers, such as the MLR, SLR and CLR, remain preserved during aging. However, multisensory cortical control mechanisms of locomotion which involves the somatosensory and parietoinsular vestibular cortices may decline with aging (Zwergal et al. 2012).

### Efferents from the cerebellum

Mori and colleagues first demonstrated that repetitive electrical stimulation applied to the mid-part of the cerebellar white matter increased the level of muscle tone (Asanome et al. 1998); identical stimuli also elicited locomotion on a treadmill (Mori et al. 1999). The locomotor-evoking site, which was defined as the CLR, corresponds to the passing fibers arising from the bilateral fastigial nuclei (FN). CLR stimulation resulted in simultaneous short-latency synaptic activation of long-descending reticulospinal and vestibulospinal cells with high synaptic security. It can be considered that the FN possesses potential capability to recruit and regulate postural- and locomotor-related subprograms which are distributed within the brainstem and spinal cord by the in-parallel activation of fastigiospinal, fastigioreticular, and fastigiovestibular pathways (Eccles et al. 1975; Mori et al. 1998, 2000).

The FN receives a copy of the output of spinal neuronal circuitry such as the CPG in addition to peripheral sensory information via spinocerebellar tracts and the vermis (Stecina et al. 2013). Activity of neurons in the FN was affected by visual, vestibular and head–trunk movements (Büttner et al. 2003). Moreover, considerable population of FN neurons whose activity was modulated by hindlimb movement received convergent vestibular inputs (McCall et al. 2015). These complex, multisensory features may permit FN neurons to rather specifically affect spinal motor functions such as coordinating postural responses during locomotion and other movements which entail changes in limb position. Neuroanatomical studies demonstrated the existence of reciprocal connections between the PPN and FN (Ruggiero et al. 1997; Hazrati and Parent 1992). Ultrastructural analysis shows that synapses formed by cerebellar fibers in PPN are of the asymmetric type that occurs predominantly on dendrites of PPN neurons, indicating that the cerebellotegmental projection is excitatory (Hazrati and Parent 1992). The connection between the PPN and cerebellum in human is established by neuroimaging studies (Muthusamy et al. 2007).

The FN of the cerebellum may, therefore, send highly integrated bodily information during ongoing movements to the posture-gait related areas in the brainstem and the cerebral cortex via the thalamus (Cavdar et al. 2002). A study using diffusion tensor imaging revealed the connection between the cerebellum and the PPN in PD patients without freezing of gait. However, freezers of patients in PD showed the absence of cerebellotegmental connectivity and increased visibility of the decussation of corticopontine fibers in the anterior pons (Schweder et al. 2010). These findings highlight the importance of corticopontine-cerebellar pathways in the pathophysiology of gait when the cerebellotegmental connection that may contribute to automatic execution of gait control is damaged in freezers of PD.

## Concluding thoughts

At the Robert Wertenberg Lecture in 1981, Marsden (1982) hypothesized that the basal ganglia are responsible for the automatic execution of learned motor plans. While walking activities in daily life are seemingly automatically performed, adaptable gait control is acquired by a traditional practice as a process of procedural learning. The procedural learning, which is acquisition of daily custom, requires the cooperative activities between motor and cognitive cortical loops with the basal ganglia and the cerebellum (Hikosaka et al. 1999; Hoshi and Tanji 2007; Middleton and Strick 2000). The motor loop is responsible for automatic execution of gait control, whereas the cognitive loop contributes to the intentional aspect of gait performance. The disturbance of motor loops in patients with PD may be one of pathophysiological mechanisms of freezing of gait (Fling et al. 2014; Schweder et al. 2010). This may further require the patients to use cognitive loops so that new networks would be reconstructed to achieve adaptive gait control. A failure in the reconstruction of appropriate networks, or mal adaptive neural compensations, may also cause posture-gait disturbances such as freezing and dystonia (Coffman et al. 2011; Fling et al. 2014; Schweder et al. 2010). It should be noted that both loops eventually activate motor systems descending from the brainstem to the spinal cord (Takakusaki 2013), indicating that the pathophysiologically produced activities in the forebrain as well as the cerebellum express disturbances of posture and gait through the activation of the brainstem-spinal motor systems.

Recent progress in neuroimaging researches in non-human primates as well as human being have poured attention into the function of the brainstem, because the mesencephalic part of the brainstem has also been recognized as one of the pathological sites that cause posture-gait disorders such as PD and progressive supranuclear palsy (Benarroch 2013; Hirsch et al. 1987; Jellinger 1988). As discussed in this review, the function of the brainstem is far more complicated than we have presumed. Specifically, reticulospinal system has multifaceted functions (Chase 1980), and particular function can be selected to perform the demanding task. The selection may depend on various factors such as the vigilance state (sleep and wakefulness) of animals, neurotransmitters acting on reticular formation and efferents to the reticular formation from the higher motor centers. Accordingly, importance of the basic researches that focused on the structures and function of the brainstem, specifically the mesencephalic control of the reticulospinal systems, should be more highlighted to understand the pathophysiological mechanisms of abnormality in posture and gait and to consider new therapeutic strategies for patients with posture-gait disorders.

## Abbreviations

ACh: Acetylcholine
BF: Basal forebrain
CLR: Cerebellar locomotor region
CPG: Central pattern generator
CS: Nucleus centralis superior
CV: Conduction velocity
DA: Dopamine
DOPA: L-3,4-dihydroxyphenylalanine
DR: Dorsal raphe nucleus
E: Extensor motoneurons
EMG: Electromyogram
EOG: Electrooculogram
EPn: Entopeduncular nucleus
EPSPs: Excitatory postsynaptic potentials
F: Flexor motoneurons
FN: Fastigial nucleus
GABA: Gamma-amino-butyric acid
GPi: Internal segment of globus pallidus
IC: Inferior colliculus
IO: Inferior olive
IPSP: Inhibitory postsynaptic potentials
LC: Locus coeruleus
LDT: Laterodorsal tegmental nucleus
LG-S: Lateral gastrocnemius-soleus
LL: Lateral lemniscus
MLF: Medial longitudinal fasciculus
MLR: Midbrain or mesencephalic locomotor region
MPTP: 1-Methyl-4-phenyl-1,2,3,6-tetrahydropyridine
MRF: Medullary reticular formation
M1: Primary motor cortex
NA: Noradrenaline
NRGc: Nucleus reticularis gigantocellularis
NRMc: Nucleus reticularis magnocellularis
NRPo: Nucleus reticularis pontis oralis
NRPc: Nucleus reticularis pontis caudalis
P: Pyramidal tract
PM: Premotor area
PMRF: Pontomedullary reticular formation
PPN: Pedunculopontine tegmental nucleus
PRF: Pontine reticular formation
PSPs: Postsynaptic potentials
REM: Rapid eye movement
RSN: Reticulospinal neuron
RN: Raphe nuclei
RM: Nucleus raphe magnus
RPa: Nucleus raphe pallidus
SCP: Superior cerebellar peduncle
SD: Standard deviation
SLR: Subthalamic locomotor region
SMA: Supplementary motor areas
SNr: Substantia nigra pars reticulata
SO: Superior olive
STN: Subthalamic nucleus
TB: Trapezoid body
VM: Medial vestibular nucleus
5-HT: Serotonin
5ST: Spinal trigeminal tract
